# Model description of combined numerical and stochastic groundwater flow in Bandung-Soreang Groundwater Basin, West Java, Indonesia

**DOI:** 10.1016/j.mex.2024.103112

**Published:** 2024-12-18

**Authors:** Achmad Darul, Dasapta Erwin Irawan, Prihadi Sumintadireja, Gumilar Ramadhan

**Affiliations:** a Doctoral Program of Geological Engineering, Faculty of Earth Sciences and Technology, Bandung Institute of Technology (ITB), Bandung, West Java 40132, Indonesia; b Applied Geology Research Group, Faculty of Earth Sciences and Technology, Bandung Institute of Technology (ITB), Bandung, West Java 40132, Indonesia; c Department of Geological Engineering, Faculty of Industrial Technology, Sumatera Institute of Technology (ITERA), Way Hui, Lampung 35365, Indonesia

**Keywords:** Stochastic model, Numeric model, Bandung-Soreang Groundwater Basin, West Java, Indonesia, Urban Hydrogeology, Stochastic approach;Ordinary kriging;Finite difference method

## Abstract

The first groundwater modeling of the Bandung Aquifer Basin in 2009 used a finite difference method with a 0.5 km² grid, representing three volcanic geological layers. It assumed uniform hydraulic properties and used an equivalent homogeneous aquifer with anisotropic hydraulic conductivity.

• *Dataset:* The study analyzed a comprehensive dataset of 196 boreholes. Of these, 130 boreholes had pumping test data, while the remaining 66 had both pumping test and well logging data. This diverse dataset provided a robust foundation for the analysis. Hydraulic conductivity (K) was estimated using the ordinary kriging method, a geostatistical technique that allows for optimal interpolation based on regression against observed values of surrounding data points.
• *Analyses:* This study examines K distribution using a block model, employing finite difference modeling with an structured grid 0.1 km². Regionalized variable scenarios were applied to reduce estimation errors for certain areas, showing improved correlation.
• *Expected output:* Results include: (1) Distribution maps of hydraulic conductivity; (2) Validation metrics and visualization comparing modeled to observed data; (3) Assessments of heterogeneous K values' impact on groundwater flow; (4) Recommendations for sustainable groundwater management practices.

## Graphical abstract

**Figure:**
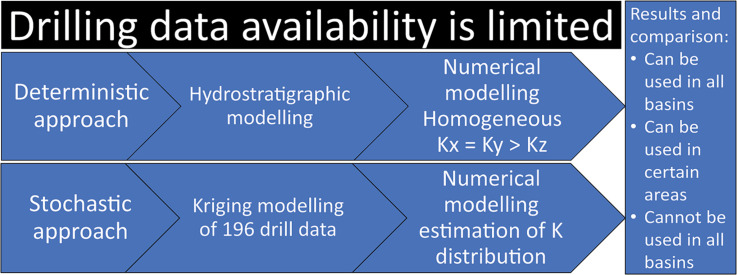

Specifications table

**Table:** 

Subject area:	Earth and Planetary Sciences
More specific subject area:	Urban Hydrogeology; Groundwater modelling
Name of your method:	Stochastic approach; Ordinary kriging; Finite difference method
Name and reference of original method:	L. Hutasoit, “Kondisi permukaan air tanah dengan dan tanpa peresapan buatan di daerah Bandung: Hasil simulasi numerik,” *Indones. J. Geosci.*, 2009, doi: 10.17014/ijog.vol4no3.20093.
Resource availability:	https://data.mendeley.com/datasets/h4tfpp7xyr/1 https://scikit-learn.org/stable/modules/generated/sklearn.model_selection.train_test_split.html https://scerf.stanford.edu/resources/software

## Background

Groundwater extraction through production wells requires careful monitoring and licensing to prevent harmful environmental impacts from overexploitation [1,2]. A case study in the Bandung Groundwater Basin illustrates this, revealing the mixing of shallow and deep groundwater. The study found a positive correlation between the mixing ratio—measured by Chlorofluorocarbon-12 concentration—and vertical flux, with the strongest correlation in the groundwater cone area. This indicates that deep groundwater is more vulnerable to contamination from shallow groundwater polluted by urban and industrial activities [3]. Another consequence of excessive groundwater extraction in the Bandung Groundwater Basin is land subsidence [4].

Groundwater modeling is crucial for understanding flow directions and interactions [5]. It plays a vital role in the treatment, recovery, and remediation of groundwater pollution. Additionally, it helps visualize the distribution of *head* (groundwater table elevation) based on hydrogeological media parameters incorporated in groundwater flow equations. These models provide significant support for policy-making [6].

Previous research on the Bandung Groundwater Basin has laid the groundwork for the current study's model setup. The conceptual model assumes an isotropic aquifer with constant vertical and horizontal hydraulic conductivity for each layer. Heterogeneity is represented by an equivalent homogeneous aquifer with anisotropic hydraulic conductivity (K), where K_x_ = K_y_ > K_z_ [7]. These earlier studies used a deterministic approach to conceptual groundwater modeling, combining direct and indirect geological observations with expert insight to create a unique model of geological heterogeneity. Hydrogeological property values were then applied based on lithostratigraphic criteria [8]. In contrast, the stochastic hydrogeology approach has revealed that the subsurface is far more heterogeneous than previously thought. Understanding this heterogeneity leads to better estimates of groundwater flow and contaminant transport, which are closely tied to the fundamental hydrogeological parameter of hydraulic conductivity [9].

The stochastic approach continues to evolve, particularly in its application to fluid flow and solute transport in hydrogeological media. It simulates the distribution of hydraulic conductivity values that govern fluid flow in various geological environments, integrating this into numerical model workflows [10]. This research aims to estimate the distribution of hydraulic conductivity values in a three-dimensional model block using a stochastic method with an ordinary kriging approach. This method will provide a realistic representation of geological complexity. The resulting hydraulic conductivity value distribution model will serve as input data for numerical groundwater modeling using the finite difference method. The novelty of this research lies in combining sub-regional hydraulic conductivity value models into regional ones in three-dimensional space [11].

This study explores the hypothesis that hydraulic conductivity values vary, even within a single sedimentary rock layer, affecting groundwater flow [12]. By using the finite difference method based on hydraulic conductivity value distributions derived from a stochastic approach, this groundwater modeling technique addresses uncertainty and offers an alternative to spatial simplification in conceptual models [13]. In summary, this paper has three objectives: (i) to reduce parameter uncertainty using a stochastic approach with ordinary kriging; (ii) to demonstrate the estimated value distribution of hydraulic conductivity in a three-dimensional block model; and (iii) to show the estimated value distribution of hydraulic conductivity, which can be used as input data for groundwater modeling using the finite-difference method. The results obtained using the Parameter Estimation Stochastic Technique (PEST) methodology are suitable for spatially representing the K values in calibrated groundwater flow models, thereby improving traditional practices in the application of hydraulic conductivity values.

## Method details

### Regional geology and conceptual groundwater modeling of the Bandung basin

The Bandung Basin, located in western Java, Indonesia, primarily consists of lake deposits. The research area, part of West Java province, includes Bandung Regency, West Bandung Regency, Sumedang Regency, Cimahi City, and Bandung City. Collectively, these form a metropolitan area with a population of seven million.

Physiographically, the area is known as the Bandung Zone. Volcanic highlands surround this zone, with the Bogor Zone an anticlinal structure of Neogene strata and volcanic intrusions bordering it to the north [14]. To the south, the Bandung Zone transitions into the Southern Mountain Slopes, characterized by a series of Quaternary volcanoes including Mounts Patuha, Tilu, Malabar, and Mandalawangi.

The Bandung Basin is an intra mountain depression. Its central part lies at 665 meters above sea level, encircled by Tertiary and Quaternary volcanic plains rising to 2,400 meters. Mount Halu bounds the basin to the west, Mounts Manglayang and Mandalawangi to the east, Mounts Burangrang, Tangkubanparahu, and Bukit Tunggul to the north, and Mount Malabar to the south.

**Fig. 1:**
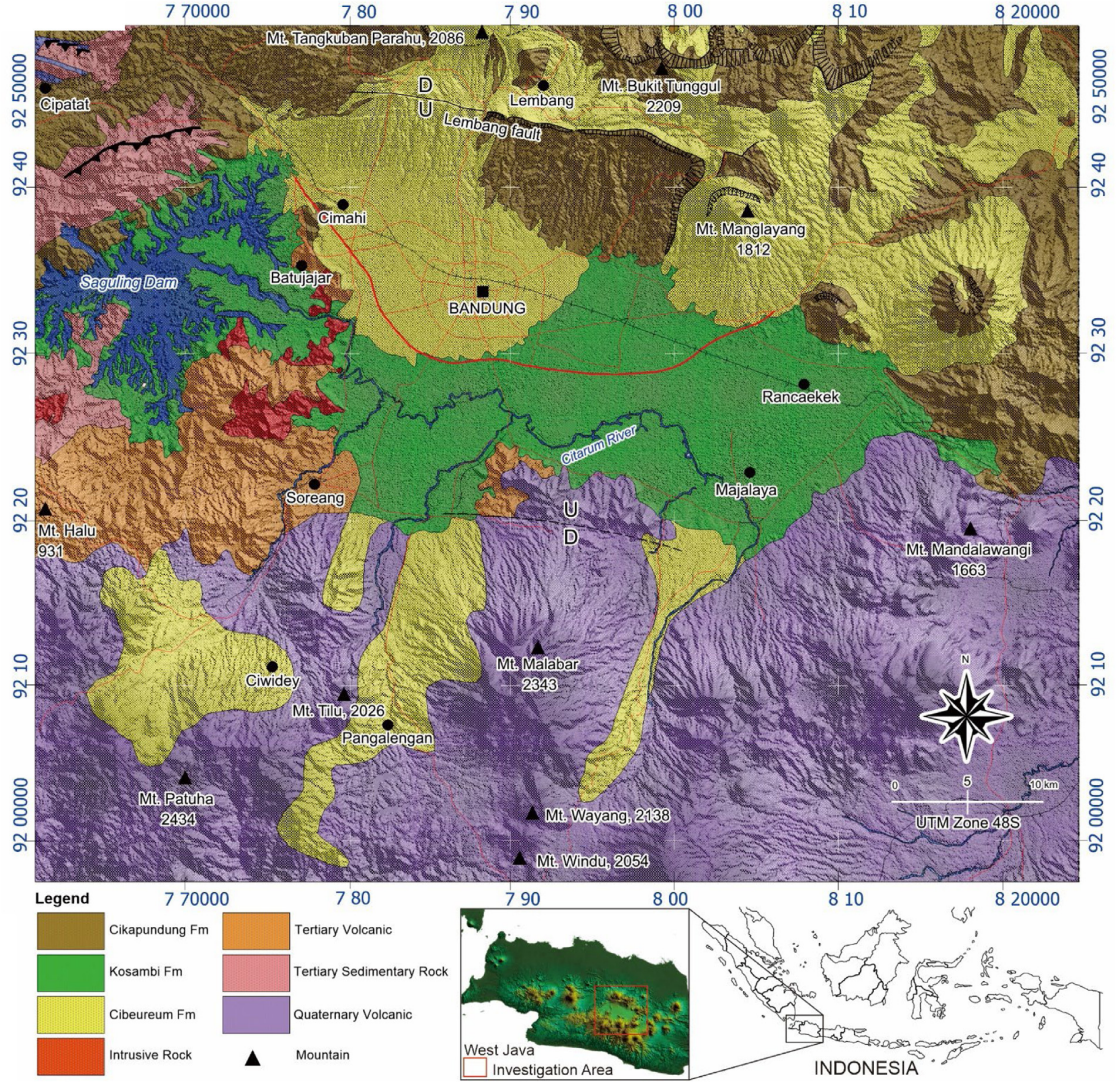
Geological map of the Bandung-Soreang Groundwater Basin study area.

The basin's central part, spanning 2,340.9 square kilometers, comprises fluvial volcaniclastic sediments, mainly thick lake deposits. The Citarum River and its tributaries constitute the area's primary drainage system (Fig. 1).

The previous finite difference conceptual model consists of six layers. Layers one (L1), two (L2), and three (L3) are designated as zone S (Shallow), representing the Kosambi Formation—a shallow groundwater system. The Kosambi Formation is an aquitard, primarily composed of claystone, siltstone, and unconsolidated sandstone from the Holocene epoch. This formation interfingers with the upper part of the Cibeureum Formation, particularly in Cimahi City and Bandung City.

**Fig. 2:**
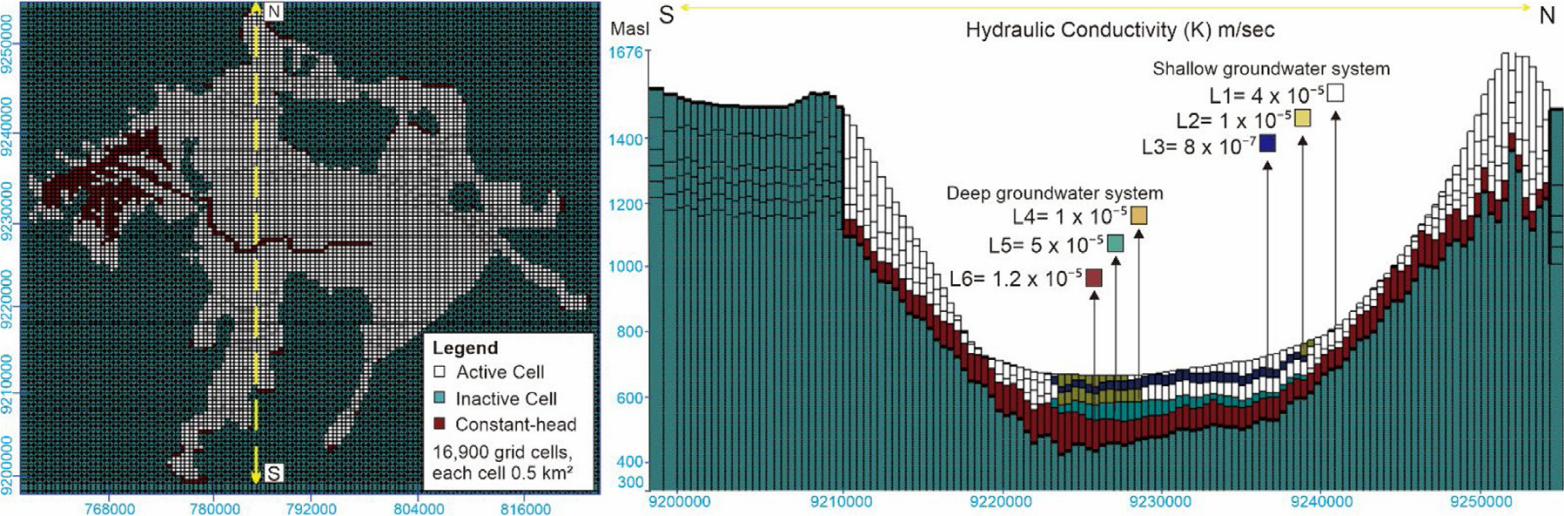
Conceptual model setup of groundwater modeling using the finite-difference method in the Bandung-Soreang Groundwater Basin and its cross-section [15].

The lower part of the Kosambi Formation, consisting of silty clay, is represented as layer three. It has a hydraulic conductivity of 8 × 10⁻⁷ meters/second and a transmissivity of 2 m²/day. The upper part, spread across the central basin, comprises claystone and sandstone. This is modeled as layer two, with a hydraulic conductivity of 1 × 10⁻⁵ meters/second and a transmissivity of 30 m²/day. Layer one represents sandy tuff from volcanic deposits on the basin slopes. It is part of the upper Cibeureum Formation, with a hydraulic conductivity of 4 × 10⁻⁵ m/second and a transmissivity of 44 m²/day (Fig. 2).

Layers four (L4), five (L5), and six (L6) are defined as zone D (Deep), which is the Cibeureum Formation a deep groundwater system. The Cibeureum Formation is the main aquifer. Its lithology consists of breccia and tuff deposits with minimal consolidation and lava intercalation from the Pleistocene-Holocene age. In the northern part, the Cibeureum Formation originates from Mount Tangkubanparahu, exhibiting a fan-shaped distribution. Some distributions also come from Mount Malabar and Mount Wayang in the south [16].

In the modeling representation, layer six comprises tuffaceous breccia, forming an aquifer with a hydraulic conductivity value of 1.2 × 10⁻⁵ meters/second and a transmissivity of 50 m²/day. Layer five, representing the Cibeureum Formation in the central part, is an aquifer composed of tuffaceous sandstone. It has a hydraulic conductivity value of 5 × 10⁻⁵ meters/second and a transmissivity of 75 m²/day. Layer four consists of sandy silt, which is confined to the central part of the basin floor. As part of the upper Cibeureum Formation, it has a hydraulic conductivity value of 1 × 10⁻⁵ meters/second and a transmissivity of 47 m²/day. The thickness of each layer in the Bandung Groundwater Basin model represents the thickness of the hydrogeological layers.

The six *layers* of zones S (*Shallow*) and D (*Deep*) are underlain by bedrock—the Cikapundung Formation. This formation's lithology consists of conglomerate, breccia, tuff, and dense andesitic lava, estimated to be of Early Pleistocene age [17]. Other units forming the bedrock include Tertiary volcanic rocks, Tertiary sedimentary rocks, and intrusive rocks, as shown on the geological map. The Cikapundung Formation bedrock is set as a no-flow boundary. In this model, the boundary condition for the Cibeureum Formation is *no flow* (Neumann type) at two locations: the contact between the aquifer and the bedrock, and the water divide in the northern part (the area of Mount Tangkubanparahu - Mount Manglayang).

### Stochastic ordinary kriging approach

**Fig. 3:**
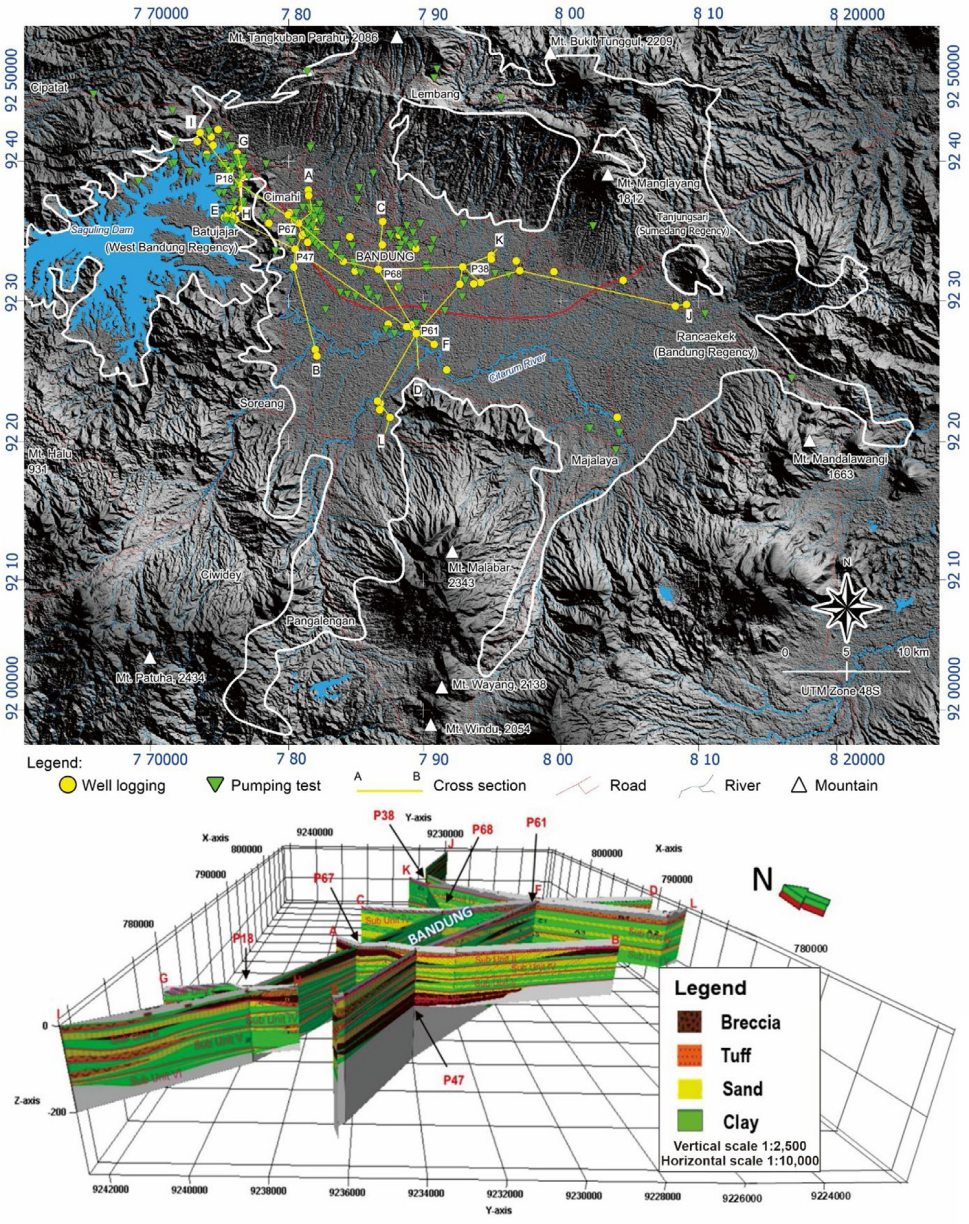
Map of pumping test locations within the Bandung-Soreang Groundwater Basin (white line), and its Hydrostratigraphic cross-section based on well logging data [19].

The arithmetic mean value of hydraulic conductivity (K) was obtained from 196 deep wells using pumping tests conducted by the Hantush-Bierschenk method [18]. Out of these wells, 66 also had well log data (Fig. 3). The well screens were positioned at various depths, ranging from 500 to 700 meters above sea level, with the screen lengths varying around 25.30 meters.

Surface elevation references are derived from the National Digital Elevation Model (DEMNAS), covering sheets 1209-31, 32, 33, 34; 1209-22, 24; and 1208-54, 63, 64. The DEMNAS satellite imagery data is compiled from multiple sources, including IFSAR, TERRASAR-X, and ALOS PALSAR data. This National Digital Elevation Model is freely accessible and can be downloaded from https://data.mendeley.com/datasets/h4tfpp7xyr/1

Well data distributed across the urban area of the Bandung Basin requires interpolation using a geostatistical method to set up a conceptual model of hydraulic conductivity (K) value distribution. This method should not rely on population averages and should be capable of processing stochastic data by forming a semivariogram.

For spatial analysis of borehole data related to K values, the ordinary kriging approach is employed [20]. Kriging, a geostatistical method, estimates a regionalized variable by treating the analyzed data as a realization of a random variable that forms a random function, using a variogram model [21]. The variogram is a statistical tool that describes, models, and explains spatial correlation between observations [22], as expressed in Eq. (1).

(1) $$\begin{array}{cc} 2\gamma \left(h\right) & =Var(\vec{Z}\left(s+h\right)-\vec{Z}\left(s\right)\\ & =E[{\left(\vec{Z}\left(s+h\right)-\vec{Z}\left(s\right)\right]}^{2} \end{array}$$

where γ(h) is the semivariogram. The semivariogram in Eq. (1) is called the theoretical variogram. When observation data is available, an experimental semivariogram is obtained. The formula for the experimental semivariogram is written in Eq. (2).

(2) $$\hat{\gamma }\left(h\right)=\frac{1}{2N\left(h\right)}\sum_{i=1}^{N\left(h\right)}[{\left(Z\left(s_{i}+h\right)-Z\left(s_{i}\right)\right]}^{2}\;$$

where $s_{i}$ represents sample locations, $Z(s_{i})$ denotes the observation value at location $s_{i}$, $\hat{\gamma }\left(h\right)$ is the experimental semivariogram at distance h, and N(h) indicates the number of data pairs $\left(s_{i},\;s_{i}+h\right)$ at distance h. The kriging method estimates the magnitude of the characteristic value $\hat{Z}$ at an unsampled point using information from surrounding sampled points Z, accounting for spatial correlation in the data. Kriging is performed after calculating a theoretical semivariogram that corresponds to the experimental semivariogram. The kriging estimator $\hat{Z}\left(s\right)$ of Z(s) is expressed as follows [23].

(3) $$\hat{Z}\left(s\right)-m\left(s\right)=\sum_{i=1}^{n}\;\lambda_{i}\left[Z\left(s_{i}\right)-m\left(s_{i}\right)\right]$$

where s and $s_{i}$ are the estimation location and nearby data points (i), respectively. m(s) is the expected value of Z(s). $\lambda_{i}$ is the weight factor, and n is the number of estimation samples. Z(s) is considered a random field with a trend component m(s) and a residual component e(s)=Z(s)−m(s). The kriging estimate for the residual at s is the sum of the weighted residuals around the data points. The value of $\lambda_{i}$ is derived from the covariance function and semivariogram, which characterize the residual component. The goal of kriging is to determine the weights that minimize the variance of the estimator, which is expressed in the following Eq. (4).

(4) $${\hat{\sigma }}_{e}^{2}=var\left\{\hat{Z}\left(s\right)-\;Z\left(s\right)\right\}$$

Ordinary Kriging is a kriging method [24] that obtains the best linear unbiased estimator (Best Linear Unbiased Estimator-BLUE). The kriging estimator $\hat{Z}\left(s_{0}\right)$ at location $s_{0}$ is a linear combination of n regional variables ($Z\left(s_{1}\right),\;Z\left(s_{2}\right),\ldots ,\;Z\left(s_{n}\right),\;$formulated in Eq. 5.

$$\hat{Z}\left(s_{0}\right)\;=\;\lambda_{1}Z\left(s_{1}\right)+\;\lambda_{2}Z\left(s_{2}\right)+\cdots +\lambda_{n}Z\left(s_{n}\right)$$

(5) $$\;=\sum_{i=1}^{n}\lambda_{i}Z\left(s_{1}\right)$$

where $\lambda_{i}$ is the kriging weight. These kriging weights are chosen so that the estimator satisfies unbiased properties, the expected value of the error at $s_{0}$ is as in Eq. 6.

$$E\left[e\left(s_{0}\right)\right]=E\left[\sum_{i=1}^{n}\lambda_{i}Z\left(s_{i}\right)-Z\left(s_{i}\right)\right]$$

(6) $$\;=\sum_{i=1}^{n}\lambda_{i}E\left[Z\left(s_{i}\right)\right]-E\left[Z\left(s_{0}\right)\right]$$

The condition for an unbiased estimator is $E[e(s_{0})]=0$ and it satisfies second-order stationarity, which is E[Z(s)]=m≠0,∀s, so Eq. 6 becomes Eq. 7 below

$$\sum_{i=1}^{n}\lambda_{i}E\left[Z\left(s_{i}\right)\right]-E\left[Z\left(s_{0}\right)\right]=0$$

$$\lambda_{1}E\left[Z\left(s_{1}\right)\right]+\lambda_{2}E[Z\left(s_{2}\right)+\cdots +\lambda_{n}E[Z\left(s_{n}\right)-E\left[Z\left(s_{0}\right)\right]=0$$

$$E\left[Z\left(s_{1}\right)\right]\lambda_{1}+E\left[Z\left(s_{2}\right)\right]\lambda_{2}+\cdots \;E\left[Z\left(s_{n}\right)]\lambda_{n}-E[Z\left(s_{0}\right)\right]=0$$

$$E\left[Z\left(s_{i}\right)\right]\sum_{i=1}^{n}\lambda_{i}-E\left[Z\left(s\right)\right]=0$$

(7) $$E\left[Z\left(s\right)\right]\left[\sum_{i=1}^{n}\lambda_{i}-1\right]=0$$

Eq. 7 is only obtained if $\sum_{i=1}^{n}=1$ (the sum of kriging weights equals 1). Based on this, $\hat{Z}\left(s_{0}\right)$ is an unbiased estimator if and only if $\sum_{i=1}^{n}=1$. In addition to satisfying the unbiased properties, it also has another property, namely minimum error variance. The error variance $\sigma_{e}^{2}$ is $var\left[e(s_{0})\right]=E{\left[\hat{Z}\left(s_{0}\right)-\;Z\left(s\right)\right]}^{2}$

$${\left[\hat{Z}\left(s_{0}\right)-Z\left(s\right)\right]}^{2}={\left[\sum_{i=1}^{n}\lambda_{i}Z\left(s_{i}\right)-\;Z\left(s_{0}\right)\right]}^{2}$$

$$={\left[\sum_{i=1}^{n}\lambda_{i}Z\left(s_{i}\right)\right]}^{2}-2Z\left(s_{0}\right)\;\sum_{i=1}^{n}\lambda_{i}Z\left(s_{i}\right)+\;Z{\left(s_{0}\right)}^{2}$$

$$=\sum_{i=1}^{n}\sum_{j=1}^{n}\lambda_{i}\lambda_{j}Z\left(s_{i}\right)Z\left(s_{j}\right)-2Z\left(s_{0}\right)\;\sum_{i=1}^{n}\lambda_{i}Z\left(s_{i}\right)+\;Z{\left(s_{0}\right)}^{2}$$

$$=\sum_{i=1}^{n}\sum_{j=1}^{n}\lambda_{i}\lambda_{j}Z\left(s_{i}\right)Z\left(s_{j}\right)-2Z\left(s_{0}\right)\sum_{i=1}^{n}\lambda_{i}Z\left(s_{i}\right)+Z{\left(s_{0}\right)}^{2}\sum_{i=1}^{n}\lambda_{i}+\sum_{i=1}^{n}\lambda_{i}Z{\left(s_{i}\right)}^{2}-\sum_{i=1}^{n}\lambda_{i}Z{\left(s_{i}\right)}^{2}$$

$$=\left[Z{\left(s_{0}\right)}^{2}\sum_{i=1}^{n}\lambda_{i}-2Z\left(s_{0}\right)\sum_{i=1}^{n}\lambda_{i}Z\left(s_{i}\right)+\sum_{i=1}^{n}\lambda_{i}Z{\left(s_{i}\right)}^{2}\;\right]-\left[\sum_{i=1}^{n}\lambda_{i}Z{\left(s_{i}\right)}^{2}-\sum_{i=1}^{n}\sum_{j=1}^{n}\lambda_{i}\lambda_{j}Z\left(s_{i}\right)Z\left(s_{j}\right)\right]$$

$$=2.\frac{1}{2}\sum_{i=1}^{n}\lambda_{i}Z{\left(s_{0}\right)}^{2}-\;2Z\left(s_{0}\right)Z\left(s_{i}\right)+Z{\left(s_{i}\right)}^{2})-\sum_{i=1}^{n}\sum_{j=1}^{n}\lambda_{i}\lambda_{j}Z{\left(s_{i}\right)}^{2}-2Z\left(s_{i}\right)-2Z\left(s_{j}\right)+Z{\left(s_{j}\right)}^{2})$$

(8) $$=2\sum_{i=1}^{n}\left[\frac{z\left(s_{0}\right)-{\left(s_{i}\right)}^{2}}{2}\right]-\sum_{j=1}^{n}\gamma_{i}\lambda_{j}\left[\frac{{\left(z\left(s_{i}\right)-z\left(s_{j}\right)\right)}^{2}}{2}\right]$$

From Eq. 8, the expectation equation is written in Eq. 9

(9) $$\begin{array}{ccc} E{\left[\hat{Z}\left(s_{0}\right)-Z\left(s_{0}\right)\right]}^{2} & = & 2\sum_{i=1}^{n}E\left[\frac{Z\left(s_{0}\right)-{\left(s_{i}\right)}^{2}}{2}\right]-\sum_{i=1}^{n}\sum_{j=1}^{n}\lambda_{i}\lambda_{j}E\left[\frac{{\left(z\left(s_{i}\right)-Z\left(s_{j}\right)\right)}^{2}}{2}\right]\\ & = & 2\sum_{i=1}^{n}\lambda_{i}\bar{\gamma }\left(s_{0,\;}s_{i}\right)-\;\sum_{i=1}^{n}\sum_{j=1}^{n}\lambda_{i}\lambda_{j}\gamma \left(s_{i},s_{j}\right) \end{array}$$

Here, $\bar{\gamma }\left(s_{0,\;}s_{i}\right)$ represents the average variogram between locations $s_{0}\;$and $s_{i}$[25]. To minimize the variance estimation while constraining the sum of the kriging weight matrix to 1, a Lagrange parameter (μ) is introduced. This results in a function as shown in Eq. 10

(10) $$f\left(s_{i}\right)=E{\left[\hat{Z}\left(s_{0}\right)-Z\left(s_{0}\right)\right]}^{2}-2\mu \left(\sum_{i=1}^{n}\lambda_{i}-1\right)$$

Function 10 is minimized by differentiating it with respect to parameters $\lambda_{1}\lambda_{2}\ldots \;\lambda_{n}$ and μ, then equating the resulting derivatives to zero. The solution is obtained in Eq. 11

$$\sum_{i=1}^{n}\lambda_{i}\gamma \left(s_{i},s_{j}\right)+\mu =\;\bar{\gamma }\left(s_{0,\;}s_{i}\right),i=1,2,\ldots ,\;n$$

(11) $$\sum_{i=1}^{n}\lambda_{i}=1$$

The minimum error variance can be determined using Lagrange multipliers with Lagrange parameters. Ordinary kriging estimates data on a semi-variogram by connecting the nearest points and determining the area of influence of the data point relationships. To constrain these relationships, grid blocks are created to cover the data distribution, effectively limiting the outermost distances.

**Fig. 4:**
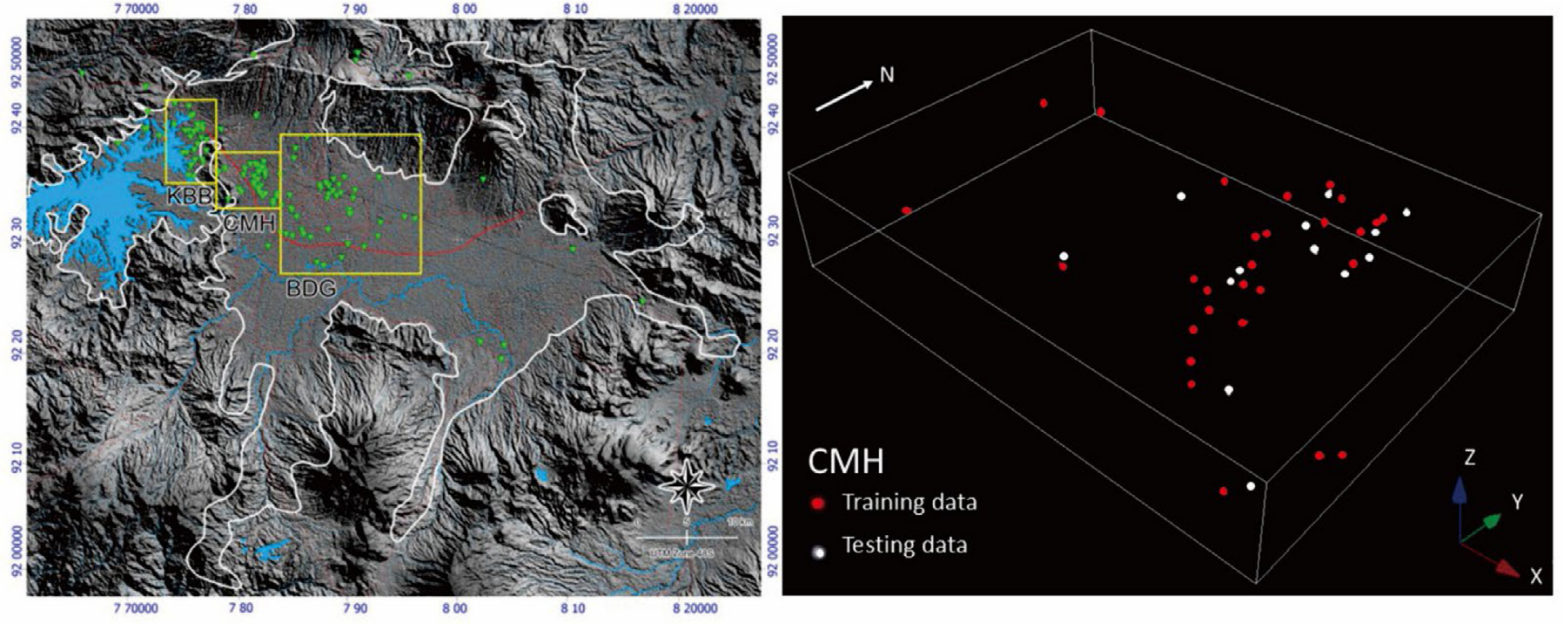
Left: Spatial distribution of borehole data for each regional variable in the Bandung-Soreang Groundwater Basin. Right: Results of the splitting for cross-validation on the grid block in Cimahi City (CMH).

Stanford Geostatistical Modeling Software-SGeMS [26], is used to analyze the distribution of hydraulic conductivity values based on borehole data. In the process of estimating the distribution of hydraulic conductivity values, clustering is applied to reduce estimation errors according to the data distribution, specifically in the regional variables of Batujajar, West Bandung Regency (KBB), Cimahi City (CMH), and Bandung City (BDG), see Fig. 4. The borehole data distribution in these three regional variable areas is divided into two datasets: 70% for training and 30% for testing, which is used for cross-validation. This percentage split is performed to evaluate the performance of the machine learning model. The data splitting stage for hydraulic conductivity from pumping tests distributed across each regional variable uses the train_test_split function from the sklearn model selection module https://scikit-learn.org/stable/modules/generated/sklearn.model_selection.train_test_split.html. Tabulation of deep well data and hydraulic conductivity values obtained from *pumping test* measurements for each well (see Table 1).

**Table 1:** Tabulated dataset of *pumping test* measurements in the Bandung -Soreang Groundwater Basin.

No.	Code of Well	Coordinate (UTM Zone 48S), Elevation DEMNAS (masl)			Depth of Well (m)	Hydraulic Conductivity m/sec	Log K	Screen depth (below ground surface in meters)					
		X	Y	Z				1	2	3	4	5	6
1	DW01	781654	9236971	719	150.00	3.18 × 10¯⁵	-4.498	66.70 - 72.80	98.10 - 104.10	-	-	-	-
3	DW03	781624	9241490	813	120.00	0.48 × 10¯⁵	-5.319	52.00 - 56.00	60.00 - 64.00	80.00 - 88.00	92.00 - 100.00	104.00 - 106.00	112.00 - 116.00
4	DW04	778096	9236345	732	130.00	3.03 × 10¯^7^	-6.519	68.00 - 76.00	80.00 - 84.00	96.00 - 100.00	112.00 - 116.00	-	-
5	DW05	782330	9235973	717	150.00	0.37 × 10¯⁵	-5.432	40.40 - 51.00	62.00 - 73.80	85.60 - 90.50	97.00 - 102.50	-	-
6	DW06	780480	9236620	730	165.00	4.41 × 10¯⁶	-5.356	112.00 - 148.00	-	-	-	-	-
7	DW07	782094	9234152	699	165.00	4.77 × 10¯⁶	-5.321	80.00 - 112.00	-	-	-	-	-
8	DW08	778698	9236630	724	105.00	7.15 × 10¯⁶	-5.146	64.00 - 68.00	72.00 - 76.00	80.00 - 84.00	88.00 - 92.00	100.00 - 104.00	-
9	DW09	783416	9234330	707	160.00	1.54 × 10¯⁵	-4.812	96.00 - 100.00	-	-	-	-	-
10	DW10	783384	9234115	705	150.00	1.68 × 10¯⁶	-5.775	60.00 - 64.00	68.00 - 88.00	-	-	-	-
11	DW11	783044	9233717	698	171.00	3.88 × 10¯⁶	-5.411	75.00 - 111.00	134.00 - 143.00	-	-	-	-
12	DW12	783012	9233440	693	160.00	2.31 × 10¯⁶	-5.636	68.00 - 76.00	80.00 - 92.00	-	-	-	-
13	DW13	778960	9233862	801	150.00	2.07 × 10¯⁶	-5.684	76.00 - 84.00	92.00 - 96.00	108.00 - 112.00	124.00 - 128.00	132.00 - 136.00	144.00 - 150.00
14	DW14	780354	9236098	721	160.00	4.27 × 10¯⁶	-5.370	84.00 - 87.00	96.00 - 99.00	105.00 - 108.00	126.00 - 129.00	-	-
15	DW15	782341	9234335	700	160.00	1.33 × 10¯⁶	-5.876	80.00 - 88.00	91.00 - 98.00	101.00 - 105.00	125.00 - 129.00	137.00 - 145.00	-
16	DW16	781424	9236005	710	120.00	8.25 × 10¯⁵	-4.084	64.30 - 68.70	76.00 - 82.00	-	-	-	-
17	DW17	781351	9235960	709	120.00	2.57 × 10¯⁵	-4.590	62.60 - 68.20	74.00 - 82.20	88.46 - 99.80	-	-	-
18	DW18	781525	9235606	705	200.00	0.21 × 10¯⁵	-5.678	88.20 - 118.80	145.00 - 163.10	-	-	-	-
19	DW19	781434	9235651	707	250.00	0.61 × 10¯⁵	-5.215	59.90 - 82.70	159.00 - 179.00	-	-	-	-
20	DW20	781417	9235506	705	200.00	3.35 × 10¯⁵	-4.475	92.00 - 98.00	100.00 - 104.00	112.00 - 116.00	123.00 - 129.00	138.00 - 144.00	156.00 - 162.00
21	DW21	781853	9234419	695	250.00	0.45 × 10¯⁵	-5.347	72.50 - 86.00	96.12 - 115.00	132.80 - 142.00	-	-	-
22	DW22	781946	9235081	702	120.00	0.23 × 10¯⁵	-5.638	79.70 - 103.80	107.90 - 118.10	-	-	-	-
23	DW23	781277	9235346	703	250.00	2.18 × 10¯⁵	-4.662	96.00 - 102.00	114.00 - 120.00	144.00 - 120.00	144.00 - 150.00	180.00 - 186.00	-
24	DW24	781676	9236337	723	144.00	1.18 × 10¯⁴	-3.928	66.00 - 96.00	-	-	-	-	-
25	DW25	782354	9236376	723	105.78	0.77 × 10¯⁵	-5.114	85.50 - 105.78	-	-	-	-	-
26	DW26	781695	9234892	701	250.00	0.10 × 10¯⁵	-6.000	68.50 - 72.00	86.12 - 105.00	-	-	-	-
27	DW27	781681	9234707	706	250.00	0.13 × 10¯⁵	-5.886	72.50 - 86.00	96.12 - 115.00	132.80 - 142.00	-	-	-
28	DW28	782278	9236206	717	150.00	6.39 × 10¯⁵	-4.194	85.00 - 90.00	97.00 - 101.00	-	-	-	-
29	DW29	780973	9237067	732	250.00	1.80 × 10¯⁶	-5.745	130.00 - 135.00	142.00 - 148.00	150.00 - 154.00	160.00 - 166.00	170.00 - 178.00	-
30	DW31	779962	9234779	706	200.00	1.62 × 10¯⁵	-4.790	84.35 - 102.53	127.33 - 146.02	158.45 - 176.05	-	-	-
31	DW32	780169	9234567	698	200.00	0.92 × 10¯⁵	-5.036	87.77 - 101.67	-	-	-	-	-
32	DW33	781664	9235663	705	200.00	0.09 × 10¯⁵	-6.046	109.00 - 130.00	149.00 - 170.00	-	-	-	-
33	DW34	782199	9236776	725	200.00	0.45 × 10¯⁵	-5.347	60.00 - 66.00	66.00 - 84.00	-	-	-	-
34	DW35	782203	9237247	732	200.00	2.36 × 10¯⁵	-4.627	68.00 - 71.00	77.00 - 80.00	101.00 - 110.00	119.00 - 122.00	125.00 - 128.00	-
35	DW36	782187	9236705	722	98.00	7.16 × 10¯⁵	-4.145	78.00 - 81.00	87.00 - 90.00	96.00 - 99.00	-	-	-
36	DW37	782142	9236789	727	108.00	1.20 × 10¯⁴	-3.921	61.00 - 64.00	77.00 - 80.00	86.00 - 89.00	107.00 - 108.00	-	-
37	DW38	781808	9236427	720	130.00	4.86 × 10¯⁶	-5.313	56.00 - 59.00	90.00 - 93.00	-	-	-	-
38	DW39	781794	9236353	716	180.00	7.99 × 10¯⁵	-4.097	122.00 - 125.00	126.00 - 132.00	-	-	-	-
39	DW40	781465	9235769	706	126.00	1.16 × 10¯⁶	-5.936	108.00 - 119.00	-	-	-	-	-
40	DW41	781457	9237058	733	166.00	0.50 × 10¯^7^	-7.301	71.24 - 82.92	126.60 - 132.31	-	-	-	-
41	DW42	781457	9237057	733	139.66	0.29 × 10¯^7^	-7.538	84.05 - 87.00	90.14 - 105.78	-	-	-	-
42	DW43	781456	9237056	733	103.96	1.58 × 10¯⁶	-5.801	81.37 - 99.28	-	-	-	-	-
43	DW44	781455	9237056	733	114.13	2.03 × 10¯⁶	-5.693	66.47 - 72.62	78.65 - 90.71	96.02 - 102.03	-	-	-
44	DW45	781996	9236787	723	200.00	0.85 × 10¯^7^	-7.071	91.23 - 113.66	119.88 - 131.44	-	-	-	-
45	DW46	781318	9235118	700	129.00	1.06 × 10¯⁵	-4.975	62.50 - 64.00	72.00 - 79.10	104.00 - 112.50	-	-	-
46	DW49	790110	9232582	700	100.00	0.49 × 10¯⁵	-5.310	56.00 - 80.00	-	-	-	-	-
47	DW50	785772	9230703	681	200.00	1.16 × 10¯⁴	-3.936	45.10 - 50.30	66.10 - 68.20	124.50 - 127.20	135.50 - 140.80	165.80 - 168.40	-
48	DW51	785820	9230637	679	250.00	6.00 × 10¯⁵	-4.222	79.50 - 84.90	108.00 - 113.30	136.40 - 142.00	170.80 - 176.40	222.50 - 225.20	229.00 - 233.00
49	DW52	789526	9235834	727	150.00	3.78 × 10¯⁵	-4.423	66.00 - 72.00	72.00 - 78.00	84.00 - 90.00	-	-	-
50	DW53	789938	9232841	692	120.00	2.08 × 10¯⁵	-4.682	61.95 - 69.45	80.91 - 88.45	-	-	-	-
51	DW54	787362	9234815	730	150.00	2.24 × 10¯⁵	-4.650	72.62 - 84.95	-	-	-	-	-
52	DW55	786130	9239495	843	120.00	1.53 × 10¯⁵	-4.815	11.20 - 47.90	-	-	-	-	-
53	DW56	788150	9235052	706	150.00	6.27 × 10¯⁵	-4.203	72.00 - 75.00	84.00 - 90.00	102.00 - 111.00	-	-	-
54	DW57	792794	9231673	677	150.00	3.48 × 10¯⁵	-4.458	78.00 - 90.00	128.00 - 145.00	-	-	-	-
55	DW58	792568	9230451	672	120.00	2.47 × 10¯⁵	-4.607	80.00 - 92.00	96.00 - 100.00	-	-	-	-
56	DW59	785624	9231042	686	95.00	1.41 × 10¯⁵	-4.851	60.00 - 64.00	68.00 - 80.00	-	-	-	-
57	DW61	784960	9237581	756	125.00	1.27 × 10¯⁵	-4.896	88.00 - 104.00	108.00 - 120.00	-	-	-	-
58	DW62	788800	9234125	702	150.00	5.66 × 10¯⁵	-4.247	66.00 - 90.00	96.00 - 120.00	126.00 - 138.00	-	-	-
59	DW63	784735	9232838	696	140.00	1.56 × 10¯⁵	-4.807	76.00 - 88.00	96.00 - 104.00	112.00 - 124.00	128.00 - 136.00	-	-
60	DW64	794955	9232169	673	150.00	0.30 × 10¯⁵	-5.523	68.00 - 76.00	88.00 - 100.00	116.00 - 128.00	136.00 - 144.00	-	-
61	DW65	785090	9238458	790	150.00	1.94 × 10¯⁵	-4.712	72.00 - 79.00	96.00 - 103.00	113.00 - 124.00	131.00 - 140.00	-	-
62	SB1	791301	9229456	673	150.00	2.16 × 10¯⁵	-4.666	84.00 - 90.00	96.00 - 102.00	105.00 - 111.00	-	-	-
63	SB2	788544	9235645	731	186.73	3.29 × 10¯⁵	-4.483	112.66 - 137.15	-	-	-	-	-
64	SB4	788112	9234291	702	125.00	1.68 × 10¯⁵	-4.775	84.00 - 96.00	100.00 - 104.00	108.00 - 116.00	-	-	-
65	SB6	790483	9234635	694	75.00	0.90 × 10¯⁶	-6.046	55.49 - 60.63	62.44 - 64.38	-	-	-	-
66	SB7	784788	9230543	675	120.00	3.40 × 10¯⁵	-4.469	79.94 - 99.44	-	-	-	-	-
67	SB8	802138	9235553	875	100.90	2.31 × 10¯⁵	-4.636	54.30 - 60.10	65.60 - 71.40	93.50 - 99.00	-	-	-
68	SB10	789952	9232508	686	80.00	6.57 × 10¯^7^	-6.182	56.00 - 80.00	-	-	-	-	-
69	SB11	786405	9230379	676	150.00	3.86 × 10¯⁵	-4.413	72.00 - 84.00	88.00 - 96.00	100.00 - 104.00	112.00 - 124.00	128.00 - 136.00	-
70	SB12	783680	9236095	732	176.00	7.47 × 10¯⁵	-4.127	105.00 - 108.00	141.00 - 147.00	162.00 - 165.00	-	-	-
71	SB13	790297	9233921	688	150.00	1.75 × 10¯⁵	-4.757	60.00 - 65.00	82.00 - 88.00	102.00 - 112.00	-	-	-
72	SB14	789015	9235153	712	100.00	1.14 × 10¯⁴	-3.943	66.00 - 72.00	72.00 - 78.00	84.00 - 90.00	-	-	-
73	SB15	787266	9235076	713	100.00	5.67 × 10¯⁶	-5.246	66.47 - 90.71	-	-	-	-	-
74	SB16	783773	9233328	699	120.00	7.87 × 10¯⁶	-5.104	72.00 - 84.00	-	-	-	-	-
75	SB17	785171	9232248	693	125.00	1.27 × 10¯⁵	-4.896	88.00 - 104.00	108.00 - 120.00	-	-	-	-
76	SB18	784193	9230679	677	120.00	1.99 × 10¯⁵	-4.701	70.00 - 76.00	79.00 - 91.00	103 - 109	-	-	-
77	SB19	783668	9230829	677	120.00	1.23 × 10¯⁵	-4.910	70.00 - 76.00	79.00 - 91.00	103 - 109	112.00 - 115.00	-	-
78	SB20	784556	9234202	716	120.00	1.00 × 10¯⁵	-5.000	51.0 - 62.40	70.30 - 77.90	81.70 - 92.40	-	-	-
79	SB21	789804	9229730	670	120.00	0.40 × 10¯⁵	-5.398	72.00 - 76.00	90.00 - 94.00	-	-	-	-
80	SB22	789804	9229730	670	120.00	0.42 × 10¯⁵	-5.377	68.00 - 72.00	87.00 - 91.00	-	-	-	-
81	SB24	789189	9234354	696	99.44	1.20 × 10¯⁵	-4.921	79.94 - 99.44	-	-	-	-	-
82	SB25	784641	9233029	699	128.00	2.62 × 10¯^7^	-6.582	72.00 - 108.00	116.00 - 128.00	-	-	-	-
83	SB27	787864	9235325	714	100.00	2.39 × 10¯⁵	-4.622	48.15 - 54.18	-	-	-	-	-
84	SB30	787952	9233914	714	150.00	4.04 × 10¯⁵	-4.394	55.00 - 67.21	-	-	-	-	-
85	BB01	775036	9240156	665	100.00	3.63 × 10¯⁵	-4.440	42.00 - 66.00	-	-	-	-	-
86	BB02	775577	9236812	664	210.00	3.61 × 10¯⁵	-4.442	56.50 - 62.50	68.00 - 73.80	79.40 - 85.10	105.00 - 107.80	-	-
87	BB03	776483	9239444	670	120.00	6.77 × 10¯⁵	-4.169	81.00 - 84.00	87.00 - 90.00	93.00 - 96.00	99.00 - 102.00	105.00 - 108.00	111.00 - 114.00
88	BB05	775900	9238372	659	150.00	1.49 × 10¯⁵	-4.827	32.00 - 56.00	104.00 - 108.00	120.00 - 124.00	-	-	-
89	BB06	776194	9240565	674	130.00	2.40 × 10¯⁵	-4.620	92.00 - 100.00	109.00 - 124.00	-	-	-	-
90	BB07	776189	9240233	676	120.00	1.03 × 10¯⁵	-4.987	38.40 - 45.00	51.30 - 71.00	83.70 - 93.50	-	-	-
91	BB08	778321	9240219	726	180.00	1.02 × 10¯⁵	-4.991	88.00 - 94.00	99.00 - 105.00	120.00 - 126.00	150.00 - 156.00	-	-
92	BB09	768992	9238988	660	120.00	1.40 × 10¯⁴	-3.854	62.09 - 68.12	72.00 - 76.30	-	-	-	-
93	BB10	775844	9235860	657	200.00	2.85 × 10¯⁵	-4.545	60.00 - 72.00	157.00 - 176.00	-	-	-	-
94	BB11	765751	9245363	446	70.00	0.46 × 10¯⁵	-5.337	25.50 - 65.70	-	-	-	-	-
95	BB13	776492	9240428	682	200.00	0.07 × 10¯⁵	-6.155	57.70 - 101.70	108.00 - 163.00	-	-	-	-
96	BB14	775553	9239561	663	200.00	1.97 × 10¯⁵	-4.706	140.00 - 162.00	-	-	-	-	-
97	BB16	776667	9237229	677	169.00	3.86 × 10¯⁶	-5.413	69.00 - 72.00	80.00 - 83.00	89.00 - 92.00	98.00 - 101.00	119.00 - 122.00	128.00 - 131.00
98	BB17	775390	9236044	622	152.10	1.47 × 10¯⁵	-4.833	80.00 - 88.00	96.00 - 104.00	112.00 - 120.00	124.00 - 132.00	140.00 - 148.00	-
99	BB18	795470	9244942	1214	100.00	1.57 × 10¯⁵	-4.804	80.00 - 100.00	-	-	-	-	-
100	BB19	774169	9242611	689	140.00	1.14 × 10¯⁴	-3.943	76.00 - 82.00	86.00 - 90.00	104.00 - 108.00	-	-	-
101	BB20	775612	9241462	664	165.00	4.16 × 10¯⁶	-5.381	56.00 - 68.00	-	-	-	-	-
102	BB21	775913	9237428	662	200.00	1.65 × 10¯⁶	-5.783	118.85 - 138.43	-	-	-	-	-
103	BB22	775396	9240142	659	200.00	3.50 × 10¯⁵	-4.456	42.00 - 66.00	-	-	-	-	-
104	BB26	776635	9240378	679	180.00	0.89 × 10¯⁶	-6.051	76.00 - 82.00	88.00 - 93.00	99.00 - 105.00	120.00 - 126.00	150.00 - 156.00	-
105	BB27	776524	9239463	673	120.00	0.81 × 10¯⁶	-6.092	76.30 - 100.70	-	-	-	-	-
106	BB28	776191	9240675	676	150.00	1.31 × 10¯⁴	-3.883	56.00 - 98.00	-	-	-	-	-
107	BB30	776608	9237489	667	120.00	0.18 × 10¯⁶	-6.745	84.50 - 105.78	-	-	-	-	-
108	BB31	776212	9238905	669	147.00	4.86 × 10¯⁶	-5.313	68.50 - 78.20	79.50 - 83.40	84.20 - 93.50	94.00 -103.50	106.00 - 126.00	
109	BB32	776999	9238304	680	120.00	0.97 × 10¯⁵	-5.013	82.00 - 110.00	-	-	-	-	-
110	BB33	775847	9240007	669	100.00	1.50 × 10¯⁶	-5.824	60.02 - 78.20	-	-	-	-	-
111	BB34	775645	9236978	668	114.00	0.28 × 10¯⁵	-5.553	36.00 - 42.00	42.00 - 48.00	75.00 - 78.00	90.00 - 96.00	102.00 - 108.00	-
112	BB35	775815	9241559	661	165.00	3.95 × 10¯⁵	-4.403	66.00 - 126.00	-	-	-	-	-
113	BB36	775993	9237464	656	160.00	1.09 × 10¯⁴	-3.963	84.50 - 95.30	-	-	-	-	-
114	BB37	776699	9240233	684	180.00	1.88 × 10¯⁵	-4.726	88.00 - 93.00	99.00 - 105.00	120.00 - 126.00	150.00 - 156.00	-	-
115	BB41	774071	9240603	656	150.00	1.38 × 10¯⁴	-3.860	66.00 - 69.00	75.00 - 72.00	87.00 - 84.00	108.00 - 102.00	114.00 - 111.00	-
116	BB45	776631	9240667	684	100.00	2.76 × 10¯⁵	-4.559	43.00 - 51.00	59.00 - 79.00	-	-	-	-
117	BB47	775220	9238183	651	120.00	1.96 × 10¯⁵	-4.708	84.00 - 96.00	112.00 - 116.00	-	-	-	-
118	DW66	772737	9239691	658	150.00	6.98 × 10¯⁵	-4.155	72.00 - 75.00	84.00 - 87.00	99.00 - 105.00	132.00 - 138.00	-	-
119	BB48	771414	9240285	662	150.00	4.97 × 10¯⁵	-4.304	72.00 - 78.00	87.00 - 90.00	102.00 - 105.00	126.00 - 129.00	135.00 - 144.00	-
120	BB49	771684	9241873	707	150.00	4.93 × 10¯⁵	-4.307	81.00 - 84.00	102.00 - 108.00	126.00 - 132.00	135.00 - 144.00	-	-
121	BB50	771620	9240339	657	150.00	9.83 × 10¯⁵	-4.007	87.00 - 93.00	117.00 - 123.00	129.00 - 135.00	138.00 - 144.00	-	-
122	DH01	782583	9229585	671	100.00	5.05 × 10¯⁵	-4.297	56.60 - 60.20	64.00 - 83.30	-	-	-	-
123	DH02	789217	9228523	661	200.00	3.57 × 10¯⁵	-4.447	106.00 - 110.00	116.00 - 122.00	124.00 - 127.00	171.00 - 178.00	-	-
124	DH03	787594	9227809	664	200.00	0.55 × 10¯⁵	-5.260	102.00 - 105.00	112.00 - 115.00	122.00 - 131.00	168.00 - 171.00	186.00 - 189.00	-
125	DH04	801737	9220788	664	180.00	9.95 × 10¯⁶	-5.002	53.30 - 59.30	65.30 - 71.30	83.30 - 89.30	95.30 - 101.30	113.30 - 119.30	125.30 - 137.30
126	DH05	801816	9220806	670	180.00	1.77 × 10¯⁵	-4.752	53.20 - 59.20	65.20 - 71.20	83.20 - 89.20	95.20 - 101.20	113.20 - 119.20	125.20 - 137.20
127	DH06	803686	9219214	681	230.00	1.97 × 10¯⁵	-4.706	60.00 - 66.00	84.00 - 90.00	96.00 - 102.00	108.00 - 114.00	120.00 - 126.00	132.00 - 138.00
128	DH07	786984	9228093	666	200.00	7.12 × 10¯⁵	-4.148	60.20 - 63.30	72.10 - 75.20	96.50 - 99.70	126.80 - 129.80	138.60 - 141.30	144.20 - 147.70
129	DH08	816097	9224686	821	200.00	1.96 × 10¯⁶	-5.708	60.00 - 72.00	84.00 - 90.00	96.00 - 102.00	114.00 - 120.00	138.00 - 150.00	168.00 - 174.00
130	DH09	810259	9229121	678	250.00	9.65 × 10¯⁵	-4.015	84.00 - 90.00	93.00 - 96.00	106.00 - 111.00	-	-	-

This process ensures reproducibility and proceeds as follows.

from sklearn.model_selection import train_test_split

from pathlib import path

import pandas as pd

file_path = Path(r"C:\Users\Darul\Dropbox\Disertasi_CAT\CAT")

filename = file_path / "pumping_KBB.xlsx"

data = pd.read_excel(filename, index_col=0)

train, test = train_test_split(data, test_size=0.3)

train.to_excel(file_path/"pumping_KBB_train.xlsx")

test.to_excel(file_path/"pumping_KBB_train.xlsx")

## Method validation

This study estimates hydraulic conductivity distribution based on pumping test data for groundwater modeling and finite difference numerical simulation. There is spatial data sparsity in Lembang, Rancaekek, and Majalaya. Borehole screen positions range from 500 to 700 meters above sea level, with testing focused on the *confined aquifer* of the Cibeureum Formation. Data distribution is random and doesn't cover entire areas. To reduce error in hydraulic conductivity value distribution estimation, data is converted to log K, then antilogged to obtain actual distribution values. Simulation uses regionalized variables based on variograms from paired sample points.

**Fig 5:**
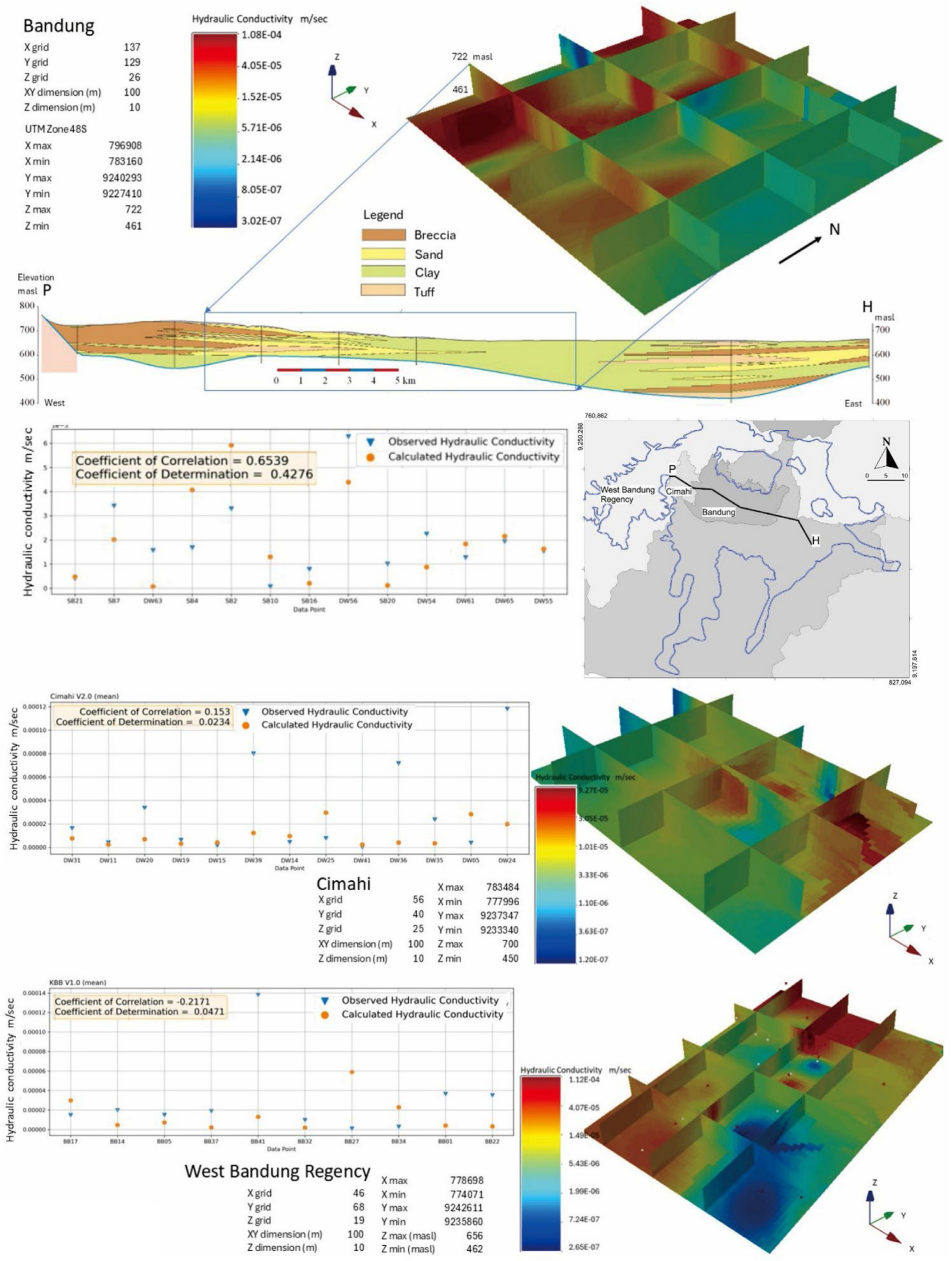
Block model of hydraulic conductivity (K) value distribution estimation and its validation for each regionalized variable.

The Bandung City area has 43 *pumping test* data points (variogram: azimuth 0, dip 5, rake 0), yielding 459,458 new estimates: 137 X, 129 Y, and 26 Z coordinates. Mean percent error: 11.24, correlation coefficient: 0.6539, determination coefficient: 0.4276. Cimahi City's industrial zone has 43 data points (variogram: azimuth 0, dip 0, rake 1), producing 48,401 new estimates: 56 X, 40 Y, and 25 Z coordinates. Mean percent error: 8.92, correlation coefficient: 0.153, determination coefficient: 0.0234. Batujajar industrial area in West Bandung Regency has 32 data points (variogram: azimuth 0, dip 0, rake 1), generating 63,841 new estimates: 46 X, 68 Y, and 19 Z coordinates. Mean percent error: 10.94, correlation coefficient: -0.2171, determination coefficient: 0.0471. The correlation coefficient, ranging from -1.0 to 1.0, indicates the relationship between model and observed data. A value near 0 suggests no linear relationship. Variogram directions align with the hydrostratigraphic cross-section from 66 *well logging* data points, previously analyzed deterministically. The hydraulic conductivity estimation model, compared with rock lithology in geological cross-sections, shows corresponding distribution patterns. Fig. 5 illustrates the K value distribution block model and its validation

The *ordinary kriging* geostatistical approach produces a hydraulic conductivity distribution model with full coordinates for each grid block. Results are expressed as histograms, representing relationships based on individual measurements. Unaccounted data is assigned a value of 0 or shown in gray. In conclusion, the stochastic estimation of hydraulic conductivity from deep well data, simulated using *ordinary kriging* and cross-validated, reveals diverse values even within single sedimentary layers, influencing groundwater flow. Table 2 presents statistical calculations, while Table 3 shows a partial subset of data points from each regionalized variable.

**Table 2:** Statistical results for each regionalized variable.

No.	Parameter	Result Statistics		West Bandung Regency
		Bandung	Cimahi	
1	Observed Mean	1.96E-05	2.84E-05	2.91E-05
2	Observed Standard Deviation	1.63E-05	3.76E-05	4.00E-05
3	Observed Variance	2.64E-10	1.42E-09	1.60E-09
4	Calculated Mean	1.93E-05	1.03E-05	1.48E-05
5	Calculated Standard Deviation	1.83E-05	9.62E-06	1.82E-05
6	Calculated Variance	3.34E-10	9.25E-11	3.32E-10
7	Covariance	1.94E-10	5.54E-11	-1.58E-10
8	Coefficient of Correlation	0.6539	0.153	-0.2171
9	Coefficient of Determination	0.4276	0.0234	0.0471
10	Mean Error (%)	8.926155609	14.17101393	17.81605952

**Table 3:** Hydraulic conductivity estimation results for 100 × 100 × 10m grids. Sampling: 20 grids from Bandung City (459,458 data points), 16 grids from Cimahi City (48,401 data points), and 16 grids from West Bandung Regency (63,841 data points). Coordinate (UTM Zone 48S), depth below ground surface in meters.

Bandung														
No.		X		Y				Z			Krigging Log K			K
1		783160.64		9227410.309				461.0269165			-4.61061			2.45E-05
2		783261.3753		9227410.309				461.0269165			-4.59189			2.56E-05
3		783362.1106		9227410.309				461.0269165			-4.5729			2.67E-05
4		783462.8459		9227410.309				461.0269165			-4.5537			2.79E-05
5		783563.5812		9227410.309				461.0269165			-4.55746			2.77E-05
6		783664.3165		9227410.309				461.0269165			-4.53496			2.92E-05
7		783765.0518		9227410.309				461.0269165			-4.51269			3.07E-05
8		783865.7871		9227410.309				461.0269165			-4.49074			3.23E-05
9		783966.5224		9227410.309				461.0269165			-4.46918			3.39E-05
10		784067.2576		9227410.309				461.0269165			-4.44807			3.56E-05
11		784167.9929		9227410.309				461.0269165			-4.42744			3.74E-05
12		784268.7282		9227410.309				461.0269165			-4.40733			3.91E-05
13		784369.4635		9227410.309				461.0269165			-4.38774			4.10E-05
14		784470.1988		9227410.309				461.0269165			-4.36865			4.28E-05
15		784570.9341		9227410.309				461.0269165			-4.35007			4.47E-05
16		784671.6694		9227410.309				461.0269165			-4.33197			4.66E-05
17		784772.4047		9227410.309				461.0269165			-4.31438			4.85E-05
18		784873.14		9227410.309				461.0269165			-4.29732			5.04E-05
19		784973.8753		9227410.309				461.0269165			-4.28085			5.24E-05
20		785074.6106		9227410.309				461.0269165			-4.26508			5.43E-05


Cimahi														
No.	X			Y		Z			Krigging Log K			K		
1	777996			9233340		519.9185791			-5.68403			2.07E-06		
2	778097.8519			9233340		519.9185791			-5.68403			2.07E-06		
3	778199.7037			9233340		519.9185791			-5.56082			2.75E-06		
4	778301.5556			9233340		519.9185791			-5.56842			2.70E-06		
5	778403.4074			9233340		519.9185791			-5.57542			2.66E-06		
6	778505.2593			9233340		519.9185791			-5.58143			2.62E-06		
7	778607.1111			9233340		519.9185791			-5.5859			2.59E-06		
8	778708.963			9233340		519.9185791			-5.58812			2.58E-06		
9	778810.8148			9233340		519.9185791			-5.58731			2.59E-06		
10	778912.6667			9233340		519.9185791			-5.5827			2.61E-06		
11	779014.5185			9233340		519.9185791			-5.57381			2.67E-06		
12	779116.3704			9233340		519.9185791			-5.56063			2.75E-06		
13	779218.2222			9233340		519.9185791			-5.5437			2.86E-06		
14	779320.0741			9233340		519.9185791			-5.52387			2.99E-06		
15	779421.9259			9233340		519.9185791			-5.50214			3.15E-06		
16	779523.7778			9233340		519.9185791			-5.4287			3.73E-06		


West Bandung Regency														
No.			X		Y		Z			Krigging Log K			K	
1			773971		9235760		490.0913086			-4.57003			2.69E-05	
2			774073.1277		9235760		490.0913086			-4.56286			2.74E-05	
3			774175.2553		9235760		490.0913086			-4.56342			2.73E-05	
4			774277.383		9235760		490.0913086			-4.56303			2.74E-05	
5			774379.5106		9235760		490.0913086			-4.56226			2.74E-05	
6			774481.6383		9235760		490.0913086			-4.56108			2.75E-05	
7			774583.766		9235760		490.0913086			-4.55953			2.76E-05	
8			774685.8936		9235760		490.0913086			-4.55762			2.77E-05	
9			774788.0213		9235760		490.0913086			-4.55543			2.78E-05	
10			774890.1489		9235760		490.0913086			-4.5989			2.52E-05	
11			774992.2766		9235760		490.0913086			-4.58887			2.58E-05	
12			775094.4043		9235760		490.0913086			-4.57983			2.63E-05	
13			775196.5319		9235760		490.0913086			-4.57205			2.68E-05	
14			775298.6596		9235760		490.0913086			-4.56585			2.72E-05	
15			775400.7872		9235760		490.0913086			-4.56149			2.74E-05	
16			775502.9149		9235760		490.0913086			-4.55925			2.76E-05	

## Limitations

Aquifer test in multi-aquifer systems is ideally conducted using the *packer test* technique to obtain hydraulic conductivity values for each aquifer or group of aquifers at different depths. So far, the practice of drillers in Indonesia has been to place the *screen* only in the main aquifer (in this case, the Cibeureum Formation) and perform aquifer testing only on that main aquifer. As a result, all hydrogeological analyses built from this data use a single hydraulic conductivity (K) value. In this study, we also assume that the K value of each aquifer in the same well is equal to the K value obtained from the aquifer test (see Table 4).

**Table 4:** Limitations and assumptions of the hydraulic conductivity value distribution estimation.

No.	Data and field conditions	Limitations	Assumptions
1	Packer test is not conducted in aquifer testing.	We only have one K value for the multi-aquifer system.	We assume the K value of each aquifer or aquifer units is equal to the K value obtained from the aquifer test.
2	The data distribution is uneven, with the majority of regionalized variables located in the Industrial agglomeration area and the city core zone.	To reduce estimation *error*, the relationships between data points are constrained by creating *grid* blocks that encompass the data distribution, thereby limiting the outermost distance.	The azimuth and dip on the geological cross-section serve as a reference for estimating the variogram direction from the relationship between two paired sample points.
3	Most drilling data points are situated in areas of volcanic rock debris (erosion products) and alluvial deposits from river activity.	These deposits and rocks exhibit high spatial diversity and depth variation, making it challenging to identify a continuous layer across a large area at a consistent depth. The *ordinary kriging* method can only partially recognize this complex geological condition.	Ordinary kriging, as a two-point analysis method, correlates the hydraulic conductivity values of alluvial deposits from river activity and volcanic rock debris (erosion products) with measurements from the nearest points.

## Preliminary results

Based on the results of modeling the estimated distribution of hydraulic conductivity values using a stochastic approach, groundwater modeling can be performed using three-dimensional finite difference method equations. The finite difference method is a numerical technique for solving differential problems. At its core, this method breaks down complex problems into smaller, simpler parts to obtain elementary solutions. It uses interpolation functions to approximate element solutions by dividing the problem domain—whether spatial or temporal—into smaller subdomains or elements.

**Fig. 6:**
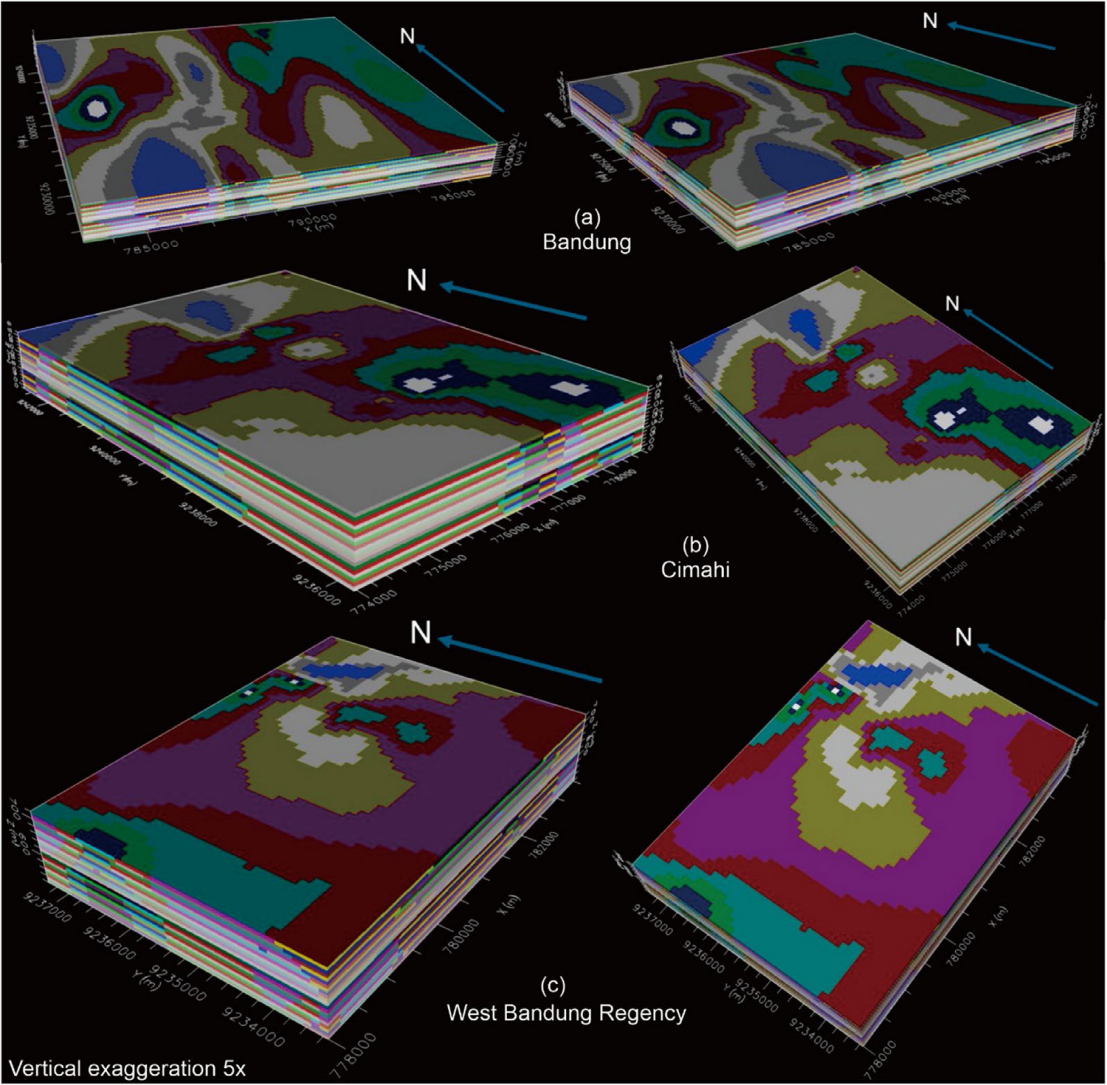
The estimated hydraulic conductivity distribution values accommodated in the finite difference method groundwater modeling software for each regionalized variable.

The finite difference approach calculates solutions for individual elements and then combines these elementary solutions. The total solution is derived by considering the continuity at the *nodes* and *interface* points. Initial results of the hydraulic conductivity value distribution estimation model, using a stochastic approach with discretization employing a *structured grid* (100 × 100 × 10meter), can be processed using the *Schlumberger Water Service* (SWS) Visual Modflow software [27]. In SWS Modflow, color contrast illustrates the adjacent hydraulic conductivity values according to the estimation model results, as depicted in Fig. 6.

## Ethics statements

This work does not use human subjects, animals, or data collected through social media as research materials

## CRediT authorship contribution statement

**Achmad Darul:** Conceptualization, Methodology, Investigation, Data curation, Formal analysis, Writing – original draft, Writing – review & editing, Visualization. **Dasapta Erwin Irawan:** Conceptualization, Methodology, Investigation, Data curation, Formal analysis, Writing – original draft, Writing – review & editing, Visualization. **Prihadi Sumintadireja:** Conceptualization, Methodology, Investigation, Data curation, Formal analysis, Writing – original draft, Writing – review & editing, Visualization. **Gumilar Ramadhan:** Conceptualization, Methodology, Investigation, Data curation, Formal analysis, Writing – original draft, Writing – review & editing, Visualization.

## Declaration of competing interest

The authors declare that they have no known competing financial interests or personal relationships that could have appeared to influence the work reported in this paper.

## Data Availability

Data will be made available on request.

## Appendix Supplementary materials

Image, application 1
