# Supplementary material for: Model description of combined numerical and stochastic groundwater flow in Bandung-Soreang Groundwater Basin, West Java, Indonesia
Source: MethodsX. 2024 Dec 18;14:103112. doi: 10.1016/j.mex.2024.103112 (PMC11732065; doi:10.1016/j.mex.2024.103112)
Supplement: Supplementary file 1 [file mmc1.docx]

Table 1. Tabulated dataset of pumping test measurements in the Bandung-Soreang Groundwater Basin.

| **No.** | **Code of Well** | **Coordinate (UTM Zone 48S), Elevation DEMNAS (masl)** | | | **Depth of Well (m)** | **Hydraulic Conductivity m/sec** | **Log K** | **Screen depth (below ground surface in meters)** | | | | | |
| --- | --- | --- | --- | --- | --- | --- | --- | --- | --- | --- | --- | --- | --- |
|  |  | **X** | **Y** | **Z** |  |  |  | **1** | **2** | **3** | **4** | **5** | **6** |
| 1 | DW01 | 781654 | 9236971 | 719 | 150.00 | 3.18 x 10¯⁵ | -4.498 | 66.70 - 72.80 | 98.10 - 104.10 | - | - | - | - |
| 3 | DW03 | 781624 | 9241490 | 813 | 120.00 | 0.48 x 10¯⁵ | -5.319 | 52.00 - 56.00 | 60.00 - 64.00 | 80.00 - 88.00 | 92.00 - 100.00 | 104.00 - 106.00 | 112.00 - 116.00 |
| 4 | DW04 | 778096 | 9236345 | 732 | 130.00 | 3.03 x 10¯^7^ | -6.519 | 68.00 - 76.00 | 80.00 - 84.00 | 96.00 - 100.00 | 112.00 - 116.00 | - | - |
| 5 | DW05 | 782330 | 9235973 | 717 | 150.00 | 0.37 x 10¯⁵ | -5.432 | 40.40 - 51.00 | 62.00 - 73.80 | 85.60 - 90.50 | 97.00 - 102.50 | - | - |
| 6 | DW06 | 780480 | 9236620 | 730 | 165.00 | 4.41 x 10¯⁶ | -5.356 | 112.00 - 148.00 | - | - | - | - | - |
| 7 | DW07 | 782094 | 9234152 | 699 | 165.00 | 4.77 x 10¯⁶ | -5.321 | 80.00 - 112.00 | - | - | - | - | - |
| 8 | DW08 | 778698 | 9236630 | 724 | 105.00 | 7.15 x 10¯⁶ | -5.146 | 64.00 - 68.00 | 72.00 - 76.00 | 80.00 - 84.00 | 88.00 - 92.00 | 100.00 - 104.00 | - |
| 9 | DW09 | 783416 | 9234330 | 707 | 160.00 | 1.54 x 10¯⁵ | -4.812 | 96.00 - 100.00 | - | - | - | - | - |
| 10 | DW10 | 783384 | 9234115 | 705 | 150.00 | 1.68 x 10¯⁶ | -5.775 | 60.00 - 64.00 | 68.00 - 88.00 | - | - | - | - |
| 11 | DW11 | 783044 | 9233717 | 698 | 171.00 | 3.88 x 10¯⁶ | -5.411 | 75.00 - 111.00 | 134.00 - 143.00 | - | - | - | - |
| 12 | DW12 | 783012 | 9233440 | 693 | 160.00 | 2.31 x 10¯⁶ | -5.636 | 68.00 - 76.00 | 80.00 - 92.00 | - | - | - | - |
| 13 | DW13 | 778960 | 9233862 | 801 | 150.00 | 2.07 x 10¯⁶ | -5.684 | 76.00 - 84.00 | 92.00 - 96.00 | 108.00 - 112.00 | 124.00 - 128.00 | 132.00 - 136.00 | 144.00 - 150.00 |
| 14 | DW14 | 780354 | 9236098 | 721 | 160.00 | 4.27 x 10¯⁶ | -5.370 | 84.00 - 87.00 | 96.00 - 99.00 | 105.00 - 108.00 | 126.00 - 129.00 | - | - |
| 15 | DW15 | 782341 | 9234335 | 700 | 160.00 | 1.33 x 10¯⁶ | -5.876 | 80.00 - 88.00 | 91.00 - 98.00 | 101.00 - 105.00 | 125.00 - 129.00 | 137.00 - 145.00 | - |
| 16 | DW16 | 781424 | 9236005 | 710 | 120.00 | 8.25 x 10¯⁵ | -4.084 | 64.30 - 68.70 | 76.00 - 82.00 | - | - | - | - |
| 17 | DW17 | 781351 | 9235960 | 709 | 120.00 | 2.57 x 10¯⁵ | -4.590 | 62.60 - 68.20 | 74.00 - 82.20 | 88.46 - 99.80 | - | - | - |
| 18 | DW18 | 781525 | 9235606 | 705 | 200.00 | 0.21 x 10¯⁵ | -5.678 | 88.20 - 118.80 | 145.00 - 163.10 | - | - | - | - |
| 19 | DW19 | 781434 | 9235651 | 707 | 250.00 | 0.61 x 10¯⁵ | -5.215 | 59.90 - 82.70 | 159.00 - 179.00 | - | - | - | - |
| 20 | DW20 | 781417 | 9235506 | 705 | 200.00 | 3.35 x 10¯⁵ | -4.475 | 92.00 - 98.00 | 100.00 - 104.00 | 112.00 - 116.00 | 123.00 - 129.00 | 138.00 - 144.00 | 156.00 - 162.00 |
| 21 | DW21 | 781853 | 9234419 | 695 | 250.00 | 0.45 x 10¯⁵ | -5.347 | 72.50 - 86.00 | 96.12 - 115.00 | 132.80 - 142.00 | - | - | - |
| 22 | DW22 | 781946 | 9235081 | 702 | 120.00 | 0.23 x 10¯⁵ | -5.638 | 79.70 - 103.80 | 107.90 - 118.10 | - | - | - | - |
| 23 | DW23 | 781277 | 9235346 | 703 | 250.00 | 2.18 x 10¯⁵ | -4.662 | 96.00 - 102.00 | 114.00 - 120.00 | 144.00 - 120.00 | 144.00 - 150.00 | 180.00 - 186.00 | - |
| 24 | DW24 | 781676 | 9236337 | 723 | 144.00 | 1.18 x 10¯⁴ | -3.928 | 66.00 - 96.00 | - | - | - | - | - |
| 25 | DW25 | 782354 | 9236376 | 723 | 105.78 | 0.77 x 10¯⁵ | -5.114 | 85.50 - 105.78 | - | - | - | - | - |
| 26 | DW26 | 781695 | 9234892 | 701 | 250.00 | 0.10 x 10¯⁵ | -6.000 | 68.50 - 72.00 | 86.12 - 105.00 | - | - | - | - |
| 27 | DW27 | 781681 | 9234707 | 706 | 250.00 | 0.13 x 10¯⁵ | -5.886 | 72.50 - 86.00 | 96.12 - 115.00 | 132.80 - 142.00 | - | - | - |
| 28 | DW28 | 782278 | 9236206 | 717 | 150.00 | 6.39 x 10¯⁵ | -4.194 | 85.00 - 90.00 | 97.00 - 101.00 | - | - | - | - |
| 29 | DW29 | 780973 | 9237067 | 732 | 250.00 | 1.80 x 10¯⁶ | -5.745 | 130.00 - 135.00 | 142.00 - 148.00 | 150.00 - 154.00 | 160.00 - 166.00 | 170.00 - 178.00 | - |
| 30 | DW31 | 779962 | 9234779 | 706 | 200.00 | 1.62 x 10¯⁵ | -4.790 | 84.35 - 102.53 | 127.33 - 146.02 | 158.45 - 176.05 | - | - | - |
| 31 | DW32 | 780169 | 9234567 | 698 | 200.00 | 0.92 x 10¯⁵ | -5.036 | 87.77 - 101.67 | - | - | - | - | - |
| 32 | DW33 | 781664 | 9235663 | 705 | 200.00 | 0.09 x 10¯⁵ | -6.046 | 109.00 - 130.00 | 149.00 - 170.00 | - | - | - | - |
| 33 | DW34 | 782199 | 9236776 | 725 | 200.00 | 0.45 x 10¯⁵ | -5.347 | 60.00 - 66.00 | 66.00 - 84.00 | - | - | - | - |
| 34 | DW35 | 782203 | 9237247 | 732 | 200.00 | 2.36 x 10¯⁵ | -4.627 | 68.00 - 71.00 | 77.00 - 80.00 | 101.00 - 110.00 | 119.00 - 122.00 | 125.00 - 128.00 | - |
| 35 | DW36 | 782187 | 9236705 | 722 | 98.00 | 7.16 x 10¯⁵ | -4.145 | 78.00 - 81.00 | 87.00 - 90.00 | 96.00 - 99.00 | - | - | - |
| 36 | DW37 | 782142 | 9236789 | 727 | 108.00 | 1.20 x 10¯⁴ | -3.921 | 61.00 - 64.00 | 77.00 - 80.00 | 86.00 - 89.00 | 107.00 - 108.00 | - | - |
| 37 | DW38 | 781808 | 9236427 | 720 | 130.00 | 4.86 x 10¯⁶ | -5.313 | 56.00 - 59.00 | 90.00 - 93.00 | - | - | - | - |
| 38 | DW39 | 781794 | 9236353 | 716 | 180.00 | 7.99 x 10¯⁵ | -4.097 | 122.00 - 125.00 | 126.00 - 132.00 | - | - | - | - |
| 39 | DW40 | 781465 | 9235769 | 706 | 126.00 | 1.16 x 10¯⁶ | -5.936 | 108.00 - 119.00 | - | - | - | - | - |
| 40 | DW41 | 781457 | 9237058 | 733 | 166.00 | 0.50 x 10¯^7^ | -7.301 | 71.24 - 82.92 | 126.60 - 132.31 | - | - | - | - |
| 41 | DW42 | 781457 | 9237057 | 733 | 139.66 | 0.29 x 10¯^7^ | -7.538 | 84.05 - 87.00 | 90.14 - 105.78 | - | - | - | - |
| 42 | DW43 | 781456 | 9237056 | 733 | 103.96 | 1.58 x 10¯⁶ | -5.801 | 81.37 - 99.28 | - | - | - | - | - |
| 43 | DW44 | 781455 | 9237056 | 733 | 114.13 | 2.03 x 10¯⁶ | -5.693 | 66.47 - 72.62 | 78.65 - 90.71 | 96.02 - 102.03 | - | - | - |
| 44 | DW45 | 781996 | 9236787 | 723 | 200.00 | 0.85 x 10¯^7^ | -7.071 | 91.23 - 113.66 | 119.88 - 131.44 | - | - | - | - |
| 45 | DW46 | 781318 | 9235118 | 700 | 129.00 | 1.06 x 10¯⁵ | -4.975 | 62.50 - 64.00 | 72.00 - 79.10 | 104.00 - 112.50 | - | - | - |
| 46 | DW49 | 790110 | 9232582 | 700 | 100.00 | 0.49 x 10¯⁵ | -5.310 | 56.00 - 80.00 | - | - | - | - | - |
| 47 | DW50 | 785772 | 9230703 | 681 | 200.00 | 1.16 x 10¯⁴ | -3.936 | 45.10 - 50.30 | 66.10 - 68.20 | 124.50 - 127.20 | 135.50 - 140.80 | 165.80 - 168.40 | - |
| 48 | DW51 | 785820 | 9230637 | 679 | 250.00 | 6.00 x 10¯⁵ | -4.222 | 79.50 - 84.90 | 108.00 - 113.30 | 136.40 - 142.00 | 170.80 - 176.40 | 222.50 - 225.20 | 229.00 - 233.00 |
| 49 | DW52 | 789526 | 9235834 | 727 | 150.00 | 3.78 x 10¯⁵ | -4.423 | 66.00 - 72.00 | 72.00 - 78.00 | 84.00 - 90.00 | - | - | - |
| 50 | DW53 | 789938 | 9232841 | 692 | 120.00 | 2.08 x 10¯⁵ | -4.682 | 61.95 - 69.45 | 80.91 - 88.45 | - | - | - | - |
| 51 | DW54 | 787362 | 9234815 | 730 | 150.00 | 2.24 x 10¯⁵ | -4.650 | 72.62 - 84.95 | - | - | - | - | - |
| 52 | DW55 | 786130 | 9239495 | 843 | 120.00 | 1.53 x 10¯⁵ | -4.815 | 11.20 - 47.90 | - | - | - | - | - |
| 53 | DW56 | 788150 | 9235052 | 706 | 150.00 | 6.27 x 10¯⁵ | -4.203 | 72.00 - 75.00 | 84.00 - 90.00 | 102.00 - 111.00 | - | - | - |
| 54 | DW57 | 792794 | 9231673 | 677 | 150.00 | 3.48 x 10¯⁵ | -4.458 | 78.00 - 90.00 | 128.00 - 145.00 | - | - | - | - |
| 55 | DW58 | 792568 | 9230451 | 672 | 120.00 | 2.47 x 10¯⁵ | -4.607 | 80.00 - 92.00 | 96.00 - 100.00 | - | - | - | - |
| 56 | DW59 | 785624 | 9231042 | 686 | 95.00 | 1.41 x 10¯⁵ | -4.851 | 60.00 - 64.00 | 68.00 - 80.00 | - | - | - | - |
| 57 | DW61 | 784960 | 9237581 | 756 | 125.00 | 1.27 x 10¯⁵ | -4.896 | 88.00 - 104.00 | 108.00 - 120.00 | - | - | - | - |
| 58 | DW62 | 788800 | 9234125 | 702 | 150.00 | 5.66 x 10¯⁵ | -4.247 | 66.00 - 90.00 | 96.00 - 120.00 | 126.00 - 138.00 | - | - | - |
| 59 | DW63 | 784735 | 9232838 | 696 | 140.00 | 1.56 x 10¯⁵ | -4.807 | 76.00 - 88.00 | 96.00 - 104.00 | 112.00 - 124.00 | 128.00 - 136.00 | - | - |
| 60 | DW64 | 794955 | 9232169 | 673 | 150.00 | 0.30 x 10¯⁵ | -5.523 | 68.00 - 76.00 | 88.00 - 100.00 | 116.00 - 128.00 | 136.00 - 144.00 | - | - |
| 61 | DW65 | 785090 | 9238458 | 790 | 150.00 | 1.94 x 10¯⁵ | -4.712 | 72.00 - 79.00 | 96.00 - 103.00 | 113.00 - 124.00 | 131.00 - 140.00 | - | - |
| 62 | SB1 | 791301 | 9229456 | 673 | 150.00 | 2.16 x 10¯⁵ | -4.666 | 84.00 - 90.00 | 96.00 - 102.00 | 105.00 - 111.00 | - | - | - |
| 63 | SB2 | 788544 | 9235645 | 731 | 186.73 | 3.29 x 10¯⁵ | -4.483 | 112.66 - 137.15 | - | - | - | - | - |
| 64 | SB4 | 788112 | 9234291 | 702 | 125.00 | 1.68 x 10¯⁵ | -4.775 | 84.00 - 96.00 | 100.00 - 104.00 | 108.00 - 116.00 | - | - | - |
| 65 | SB6 | 790483 | 9234635 | 694 | 75.00 | 0.90 x 10¯⁶ | -6.046 | 55.49 - 60.63 | 62.44 - 64.38 | - | - | - | - |
| 66 | SB7 | 784788 | 9230543 | 675 | 120.00 | 3.40 x 10¯⁵ | -4.469 | 79.94 - 99.44 | - | - | - | - | - |
| 67 | SB8 | 802138 | 9235553 | 875 | 100.90 | 2.31 x 10¯⁵ | -4.636 | 54.30 - 60.10 | 65.60 - 71.40 | 93.50 - 99.00 | - | - | - |
| 68 | SB10 | 789952 | 9232508 | 686 | 80.00 | 6.57 x 10¯^7^ | -6.182 | 56.00 - 80.00 | - | - | - | - | - |
| 69 | SB11 | 786405 | 9230379 | 676 | 150.00 | 3.86 x 10¯⁵ | -4.413 | 72.00 - 84.00 | 88.00 - 96.00 | 100.00 - 104.00 | 112.00 - 124.00 | 128.00 - 136.00 | - |
| 70 | SB12 | 783680 | 9236095 | 732 | 176.00 | 7.47 x 10¯⁵ | -4.127 | 105.00 - 108.00 | 141.00 - 147.00 | 162.00 - 165.00 | - | - | - |
| 71 | SB13 | 790297 | 9233921 | 688 | 150.00 | 1.75 x 10¯⁵ | -4.757 | 60.00 - 65.00 | 82.00 - 88.00 | 102.00 - 112.00 | - | - | - |
| 72 | SB14 | 789015 | 9235153 | 712 | 100.00 | 1.14 x 10¯⁴ | -3.943 | 66.00 - 72.00 | 72.00 - 78.00 | 84.00 - 90.00 | - | - | - |
| 73 | SB15 | 787266 | 9235076 | 713 | 100.00 | 5.67 x 10¯⁶ | -5.246 | 66.47 - 90.71 | - | - | - | - | - |
| 74 | SB16 | 783773 | 9233328 | 699 | 120.00 | 7.87 x 10¯⁶ | -5.104 | 72.00 - 84.00 | - | - | - | - | - |
| 75 | SB17 | 785171 | 9232248 | 693 | 125.00 | 1.27 x 10¯⁵ | -4.896 | 88.00 - 104.00 | 108.00 - 120.00 | - | - | - | - |
| 76 | SB18 | 784193 | 9230679 | 677 | 120.00 | 1.99 x 10¯⁵ | -4.701 | 70.00 - 76.00 | 79.00 - 91.00 | 103 - 109 | - | - | - |
| 77 | SB19 | 783668 | 9230829 | 677 | 120.00 | 1.23 x 10¯⁵ | -4.910 | 70.00 - 76.00 | 79.00 - 91.00 | 103 - 109 | 112.00 - 115.00 | - | - |
| 78 | SB20 | 784556 | 9234202 | 716 | 120.00 | 1.00 x 10¯⁵ | -5.000 | 51.0 - 62.40 | 70.30 - 77.90 | 81.70 - 92.40 | - | - | - |
| 79 | SB21 | 789804 | 9229730 | 670 | 120.00 | 0.40 x 10¯⁵ | -5.398 | 72.00 - 76.00 | 90.00 - 94.00 | - | - | - | - |
| 80 | SB22 | 789804 | 9229730 | 670 | 120.00 | 0.42 x 10¯⁵ | -5.377 | 68.00 - 72.00 | 87.00 - 91.00 | - | - | - | - |
| 81 | SB24 | 789189 | 9234354 | 696 | 99.44 | 1.20 x 10¯⁵ | -4.921 | 79.94 - 99.44 | - | - | - | - | - |
| 82 | SB25 | 784641 | 9233029 | 699 | 128.00 | 2.62 x 10¯^7^ | -6.582 | 72.00 - 108.00 | 116.00 - 128.00 | - | - | - | - |
| 83 | SB27 | 787864 | 9235325 | 714 | 100.00 | 2.39 x 10¯⁵ | -4.622 | 48.15 - 54.18 | - | - | - | - | - |
| 84 | SB30 | 787952 | 9233914 | 714 | 150.00 | 4.04 x 10¯⁵ | -4.394 | 55.00 - 67.21 | - | - | - | - | - |
| 85 | BB01 | 775036 | 9240156 | 665 | 100.00 | 3.63 x 10¯⁵ | -4.440 | 42.00 - 66.00 | - | - | - | - | - |
| 86 | BB02 | 775577 | 9236812 | 664 | 210.00 | 3.61 x 10¯⁵ | -4.442 | 56.50 - 62.50 | 68.00 - 73.80 | 79.40 - 85.10 | 105.00 - 107.80 | - | - |
| 87 | BB03 | 776483 | 9239444 | 670 | 120.00 | 6.77 x 10¯⁵ | -4.169 | 81.00 - 84.00 | 87.00 - 90.00 | 93.00 - 96.00 | 99.00 - 102.00 | 105.00 - 108.00 | 111.00 - 114.00 |
| 88 | BB05 | 775900 | 9238372 | 659 | 150.00 | 1.49 x 10¯⁵ | -4.827 | 32.00 - 56.00 | 104.00 - 108.00 | 120.00 - 124.00 | - | - | - |
| 89 | BB06 | 776194 | 9240565 | 674 | 130.00 | 2.40 x 10¯⁵ | -4.620 | 92.00 - 100.00 | 109.00 - 124.00 | - | - | - | - |
| 90 | BB07 | 776189 | 9240233 | 676 | 120.00 | 1.03 x 10¯⁵ | -4.987 | 38.40 - 45.00 | 51.30 - 71.00 | 83.70 - 93.50 | - | - | - |
| 91 | BB08 | 778321 | 9240219 | 726 | 180.00 | 1.02 x 10¯⁵ | -4.991 | 88.00 - 94.00 | 99.00 - 105.00 | 120.00 - 126.00 | 150.00 - 156.00 | - | - |
| 92 | BB09 | 768992 | 9238988 | 660 | 120.00 | 1.40 x 10¯⁴ | -3.854 | 62.09 - 68.12 | 72.00 - 76.30 | - | - | - | - |
| 93 | BB10 | 775844 | 9235860 | 657 | 200.00 | 2.85 x 10¯⁵ | -4.545 | 60.00 - 72.00 | 157.00 - 176.00 | - | - | - | - |
| 94 | BB11 | 765751 | 9245363 | 446 | 70.00 | 0.46 x 10¯⁵ | -5.337 | 25.50 - 65.70 | - | - | - | - | - |
| 95 | BB13 | 776492 | 9240428 | 682 | 200.00 | 0.07 x 10¯⁵ | -6.155 | 57.70 - 101.70 | 108.00 - 163.00 | - | - | - | - |
| 96 | BB14 | 775553 | 9239561 | 663 | 200.00 | 1.97 x 10¯⁵ | -4.706 | 140.00 - 162.00 | - | - | - | - | - |
| 97 | BB16 | 776667 | 9237229 | 677 | 169.00 | 3.86 x 10¯⁶ | -5.413 | 69.00 - 72.00 | 80.00 - 83.00 | 89.00 - 92.00 | 98.00 - 101.00 | 119.00 - 122.00 | 128.00 - 131.00 |
| 98 | BB17 | 775390 | 9236044 | 622 | 152.10 | 1.47 x 10¯⁵ | -4.833 | 80.00 - 88.00 | 96.00 - 104.00 | 112.00 - 120.00 | 124.00 - 132.00 | 140.00 - 148.00 | - |
| 99 | BB18 | 795470 | 9244942 | 1214 | 100.00 | 1.57 x 10¯⁵ | -4.804 | 80.00 - 100.00 | - | - | - | - | - |
| 100 | BB19 | 774169 | 9242611 | 689 | 140.00 | 1.14 x 10¯⁴ | -3.943 | 76.00 - 82.00 | 86.00 - 90.00 | 104.00 - 108.00 | - | - | - |
| 101 | BB20 | 775612 | 9241462 | 664 | 165.00 | 4.16 x 10¯⁶ | -5.381 | 56.00 - 68.00 | - | - | - | - | - |
| 102 | BB21 | 775913 | 9237428 | 662 | 200.00 | 1.65 x 10¯⁶ | -5.783 | 118.85 - 138.43 | - | - | - | - | - |
| 103 | BB22 | 775396 | 9240142 | 659 | 200.00 | 3.50 x 10¯⁵ | -4.456 | 42.00 - 66.00 | - | - | - | - | - |
| 104 | BB26 | 776635 | 9240378 | 679 | 180.00 | 0.89 x 10¯⁶ | -6.051 | 76.00 - 82.00 | 88.00 - 93.00 | 99.00 - 105.00 | 120.00 - 126.00 | 150.00 - 156.00 | - |
| 105 | BB27 | 776524 | 9239463 | 673 | 120.00 | 0.81 x 10¯⁶ | -6.092 | 76.30 - 100.70 | - | - | - | - | - |
| 106 | BB28 | 776191 | 9240675 | 676 | 150.00 | 1.31 x 10¯⁴ | -3.883 | 56.00 - 98.00 | - | - | - | - | - |
| 107 | BB30 | 776608 | 9237489 | 667 | 120.00 | 0.18 x 10¯⁶ | -6.745 | 84.50 - 105.78 | - | - | - | - | - |
| 108 | BB31 | 776212 | 9238905 | 669 | 147.00 | 4.86 x 10¯⁶ | -5.313 | 68.50 - 78.20 | 79.50 - 83.40 | 84.20 - 93.50 | 94.00 -103.50 | 106.00 - 126.00 |  |
| 109 | BB32 | 776999 | 9238304 | 680 | 120.00 | 0.97 x 10¯⁵ | -5.013 | 82.00 - 110.00 | - | - | - | - | - |
| 110 | BB33 | 775847 | 9240007 | 669 | 100.00 | 1.50 x 10¯⁶ | -5.824 | 60.02 - 78.20 | - | - | - | - | - |
| 111 | BB34 | 775645 | 9236978 | 668 | 114.00 | 0.28 x 10¯⁵ | -5.553 | 36.00 - 42.00 | 42.00 - 48.00 | 75.00 - 78.00 | 90.00 - 96.00 | 102.00 - 108.00 | - |
| 112 | BB35 | 775815 | 9241559 | 661 | 165.00 | 3.95 x 10¯⁵ | -4.403 | 66.00 - 126.00 | - | - | - | - | - |
| 113 | BB36 | 775993 | 9237464 | 656 | 160.00 | 1.09 x 10¯⁴ | -3.963 | 84.50 - 95.30 | - | - | - | - | - |
| 114 | BB37 | 776699 | 9240233 | 684 | 180.00 | 1.88 x 10¯⁵ | -4.726 | 88.00 - 93.00 | 99.00 - 105.00 | 120.00 - 126.00 | 150.00 - 156.00 | - | - |
| 115 | BB41 | 774071 | 9240603 | 656 | 150.00 | 1.38 x 10¯⁴ | -3.860 | 66.00 - 69.00 | 75.00 - 72.00 | 87.00 - 84.00 | 108.00 - 102.00 | 114.00 - 111.00 | - |
| 116 | BB45 | 776631 | 9240667 | 684 | 100.00 | 2.76 x 10¯⁵ | -4.559 | 43.00 - 51.00 | 59.00 - 79.00 | - | - | - | - |
| 117 | BB47 | 775220 | 9238183 | 651 | 120.00 | 1.96 x 10¯⁵ | -4.708 | 84.00 - 96.00 | 112.00 - 116.00 | - | - | - | - |
| 118 | DW66 | 772737 | 9239691 | 658 | 150.00 | 6.98 x 10¯⁵ | -4.155 | 72.00 - 75.00 | 84.00 - 87.00 | 99.00 - 105.00 | 132.00 - 138.00 | - | - |
| 119 | BB48 | 771414 | 9240285 | 662 | 150.00 | 4.97 x 10¯⁵ | -4.304 | 72.00 - 78.00 | 87.00 - 90.00 | 102.00 - 105.00 | 126.00 - 129.00 | 135.00 - 144.00 | - |
| 120 | BB49 | 771684 | 9241873 | 707 | 150.00 | 4.93 x 10¯⁵ | -4.307 | 81.00 - 84.00 | 102.00 - 108.00 | 126.00 - 132.00 | 135.00 - 144.00 | - | - |
| 121 | BB50 | 771620 | 9240339 | 657 | 150.00 | 9.83 x 10¯⁵ | -4.007 | 87.00 - 93.00 | 117.00 - 123.00 | 129.00 - 135.00 | 138.00 - 144.00 | - | - |
| 122 | DH01 | 782583 | 9229585 | 671 | 100.00 | 5.05 x 10¯⁵ | -4.297 | 56.60 - 60.20 | 64.00 - 83.30 | - | - | - | - |
| 123 | DH02 | 789217 | 9228523 | 661 | 200.00 | 3.57 x 10¯⁵ | -4.447 | 106.00 - 110.00 | 116.00 - 122.00 | 124.00 - 127.00 | 171.00 - 178.00 | - | - |
| 124 | DH03 | 787594 | 9227809 | 664 | 200.00 | 0.55 x 10¯⁵ | -5.260 | 102.00 - 105.00 | 112.00 - 115.00 | 122.00 - 131.00 | 168.00 - 171.00 | 186.00 - 189.00 | - |
| 125 | DH04 | 801737 | 9220788 | 664 | 180.00 | 9.95 x 10¯⁶ | -5.002 | 53.30 - 59.30 | 65.30 - 71.30 | 83.30 - 89.30 | 95.30 - 101.30 | 113.30 - 119.30 | 125.30 - 137.30 |
| 126 | DH05 | 801816 | 9220806 | 670 | 180.00 | 1.77 x 10¯⁵ | -4.752 | 53.20 - 59.20 | 65.20 - 71.20 | 83.20 - 89.20 | 95.20 - 101.20 | 113.20 - 119.20 | 125.20 - 137.20 |
| 127 | DH06 | 803686 | 9219214 | 681 | 230.00 | 1.97 x 10¯⁵ | -4.706 | 60.00 - 66.00 | 84.00 - 90.00 | 96.00 - 102.00 | 108.00 - 114.00 | 120.00 - 126.00 | 132.00 - 138.00 |
| 128 | DH07 | 786984 | 9228093 | 666 | 200.00 | 7.12 x 10¯⁵ | -4.148 | 60.20 - 63.30 | 72.10 - 75.20 | 96.50 - 99.70 | 126.80 - 129.80 | 138.60 - 141.30 | 144.20 - 147.70 |
| 129 | DH08 | 816097 | 9224686 | 821 | 200.00 | 1.96 x 10¯⁶ | -5.708 | 60.00 - 72.00 | 84.00 - 90.00 | 96.00 - 102.00 | 114.00 - 120.00 | 138.00 - 150.00 | 168.00 - 174.00 |
| 130 | DH09 | 810259 | 9229121 | 678 | 250.00 | 9.65 x 10¯⁵ | -4.015 | 84.00 - 90.00 | 93.00 - 96.00 | 106.00 - 111.00 | - | - | - |

Table 2. Statistical results for each regionalized variable.

|  | Result Statistics | | | |
| --- | --- | --- | --- | --- |
| No. | Parameter | Bandung | Cimahi | West Bandung Regency |
| 1 | Observed Mean | 1.96E-05 | 2.84E-05 | 2.91E-05 |
| 2 | Observed Standard Deviation | 1.63E-05 | 3.76E-05 | 4.00E-05 |
| 3 | Observed Variance | 2.64E-10 | 1.42E-09 | 1.60E-09 |
| 4 | Calculated Mean | 1.93E-05 | 1.03E-05 | 1.48E-05 |
| 5 | Calculated Standard Deviation | 1.83E-05 | 9.62E-06 | 1.82E-05 |
| 6 | Calculated Variance | 3.34E-10 | 9.25E-11 | 3.32E-10 |
| 7 | Covariance | 1.94E-10 | 5.54E-11 | -1.58E-10 |
| 8 | Coefficient of Correlation | 0.6539 | 0.153 | -0.2171 |
| 9 | Coefficient of Determination | 0.4276 | 0.0234 | 0.0471 |
| 10 | Mean Error (%) | 8.926155609 | 14.17101393 | 17.81605952 |

Table 3. Hydraulic conductivity estimation results for 100x100x10m grids. Sampling: 20 grids from Bandung City (459,458 data points), 16 grids from Cimahi City (48,401 data points), and 16 grids from West Bandung Regency (63,841 data points). Coordinate (UTM Zone 48S), depth below ground surface in meters.

| Bandung | | | | | |
| --- | --- | --- | --- | --- | --- |
| No. | X | Y | Z | Krigging Log K | K |
| 1 | 783160.64 | 9227410.309 | 461.0269165 | -4.61061 | 2.45E-05 |
| 2 | 783261.3753 | 9227410.309 | 461.0269165 | -4.59189 | 2.56E-05 |
| 3 | 783362.1106 | 9227410.309 | 461.0269165 | -4.5729 | 2.67E-05 |
| 4 | 783462.8459 | 9227410.309 | 461.0269165 | -4.5537 | 2.79E-05 |
| 5 | 783563.5812 | 9227410.309 | 461.0269165 | -4.55746 | 2.77E-05 |
| 6 | 783664.3165 | 9227410.309 | 461.0269165 | -4.53496 | 2.92E-05 |
| 7 | 783765.0518 | 9227410.309 | 461.0269165 | -4.51269 | 3.07E-05 |
| 8 | 783865.7871 | 9227410.309 | 461.0269165 | -4.49074 | 3.23E-05 |
| 9 | 783966.5224 | 9227410.309 | 461.0269165 | -4.46918 | 3.39E-05 |
| 10 | 784067.2576 | 9227410.309 | 461.0269165 | -4.44807 | 3.56E-05 |
| 11 | 784167.9929 | 9227410.309 | 461.0269165 | -4.42744 | 3.74E-05 |
| 12 | 784268.7282 | 9227410.309 | 461.0269165 | -4.40733 | 3.91E-05 |
| 13 | 784369.4635 | 9227410.309 | 461.0269165 | -4.38774 | 4.10E-05 |
| 14 | 784470.1988 | 9227410.309 | 461.0269165 | -4.36865 | 4.28E-05 |
| 15 | 784570.9341 | 9227410.309 | 461.0269165 | -4.35007 | 4.47E-05 |
| 16 | 784671.6694 | 9227410.309 | 461.0269165 | -4.33197 | 4.66E-05 |
| 17 | 784772.4047 | 9227410.309 | 461.0269165 | -4.31438 | 4.85E-05 |
| 18 | 784873.14 | 9227410.309 | 461.0269165 | -4.29732 | 5.04E-05 |
| 19 | 784973.8753 | 9227410.309 | 461.0269165 | -4.28085 | 5.24E-05 |
| 20 | 785074.6106 | 9227410.309 | 461.0269165 | -4.26508 | 5.43E-05 |

| Cimahi | | | | | |
| --- | --- | --- | --- | --- | --- |
| No. | X | Y | Z | Krigging Log K | K |
| 1 | 777996 | 9233340 | 519.9185791 | -5.68403 | 2.07E-06 |
| 2 | 778097.8519 | 9233340 | 519.9185791 | -5.68403 | 2.07E-06 |
| 3 | 778199.7037 | 9233340 | 519.9185791 | -5.56082 | 2.75E-06 |
| 4 | 778301.5556 | 9233340 | 519.9185791 | -5.56842 | 2.70E-06 |
| 5 | 778403.4074 | 9233340 | 519.9185791 | -5.57542 | 2.66E-06 |
| 6 | 778505.2593 | 9233340 | 519.9185791 | -5.58143 | 2.62E-06 |
| 7 | 778607.1111 | 9233340 | 519.9185791 | -5.5859 | 2.59E-06 |
| 8 | 778708.963 | 9233340 | 519.9185791 | -5.58812 | 2.58E-06 |
| 9 | 778810.8148 | 9233340 | 519.9185791 | -5.58731 | 2.59E-06 |
| 10 | 778912.6667 | 9233340 | 519.9185791 | -5.5827 | 2.61E-06 |
| 11 | 779014.5185 | 9233340 | 519.9185791 | -5.57381 | 2.67E-06 |
| 12 | 779116.3704 | 9233340 | 519.9185791 | -5.56063 | 2.75E-06 |
| 13 | 779218.2222 | 9233340 | 519.9185791 | -5.5437 | 2.86E-06 |
| 14 | 779320.0741 | 9233340 | 519.9185791 | -5.52387 | 2.99E-06 |
| 15 | 779421.9259 | 9233340 | 519.9185791 | -5.50214 | 3.15E-06 |
| 16 | 779523.7778 | 9233340 | 519.9185791 | -5.4287 | 3.73E-06 |

| West Bandung Regency | | | | | |
| --- | --- | --- | --- | --- | --- |
| No. | X | Y | Z | Krigging Log K | K |
| 1 | 773971 | 9235760 | 490.0913086 | -4.57003 | 2.69E-05 |
| 2 | 774073.1277 | 9235760 | 490.0913086 | -4.56286 | 2.74E-05 |
| 3 | 774175.2553 | 9235760 | 490.0913086 | -4.56342 | 2.73E-05 |
| 4 | 774277.383 | 9235760 | 490.0913086 | -4.56303 | 2.74E-05 |
| 5 | 774379.5106 | 9235760 | 490.0913086 | -4.56226 | 2.74E-05 |
| 6 | 774481.6383 | 9235760 | 490.0913086 | -4.56108 | 2.75E-05 |
| 7 | 774583.766 | 9235760 | 490.0913086 | -4.55953 | 2.76E-05 |
| 8 | 774685.8936 | 9235760 | 490.0913086 | -4.55762 | 2.77E-05 |
| 9 | 774788.0213 | 9235760 | 490.0913086 | -4.55543 | 2.78E-05 |
| 10 | 774890.1489 | 9235760 | 490.0913086 | -4.5989 | 2.52E-05 |
| 11 | 774992.2766 | 9235760 | 490.0913086 | -4.58887 | 2.58E-05 |
| 12 | 775094.4043 | 9235760 | 490.0913086 | -4.57983 | 2.63E-05 |
| 13 | 775196.5319 | 9235760 | 490.0913086 | -4.57205 | 2.68E-05 |
| 14 | 775298.6596 | 9235760 | 490.0913086 | -4.56585 | 2.72E-05 |
| 15 | 775400.7872 | 9235760 | 490.0913086 | -4.56149 | 2.74E-05 |
| 16 | 775502.9149 | 9235760 | 490.0913086 | -4.55925 | 2.76E-05 |
